# Healthcare Sector Dynamics in Turkey (2002–2022): Trends, Breakpoints, and Policy Implications (Privatization in the Hospital Sector)

**DOI:** 10.3390/healthcare13060622

**Published:** 2025-03-13

**Authors:** Erdinç Ünal, Salim Yılmaz

**Affiliations:** 1 Department of Health Management, Faculty of Health Sciences, Ardahan University, Ardahan 75000, Turkey; erdincunal@ardahan.edu.tr; 2 Department of Health Management, Faculty of Health Sciences, Acibadem Mehmet Ali Aydinlar University, Istanbul 34752, Turkey

**Keywords:** healthcare privatization, hospital sector, health sector in Turkey

## Abstract

**Background/Objectives:** This study examines the transformation of Turkey’s hospital sector from 2002 to 2022, focusing on physical capacity, service utilization, and workforce distribution in the public and private sectors. **Methods:** Longitudinal data from the Ministry of Health were analyzed using trend and breakpoint methods to evaluate hospital beds, qualified beds, intensive care beds, service volumes (outpatient visits, inpatient admissions, surgeries, and hospitalization days), and staffing (physicians, nurses, and midwives). **Results:** Findings reveal a marked shift in the balance between public and private providers. Due to public regulations effectively controlling resource allocation, the private sector’s share expanded to around one-fourth of the system. Private capacity in total beds rose from 7.53% to 21.00%, outpatient visits from 4.58% to 15.07%, and inpatient admissions from 10.10% to 30.63%. Breakpoint analyses indicate crucial turning points around 2005, 2008, and 2011, when policy changes restricted public capacity but facilitated private investment. Although the public sector’s share in total beds declined, its proportion of qualified and intensive care beds, as well as dialysis machines, increased, suggesting a strategic shift toward complex, high-quality services. **Conclusions:** Over the past 20 years, Turkey’s hospital sector exemplifies privatization without ownership transfer. Although delayed, private hospital expansion aligned with global neoliberal trends. Policy regulations played a key role in both promoting and limiting sector growth. A constant conflict exists between market-driven resource allocation and public health needs, which must be considered in restructuring efforts alongside private sector motivations.

## 1. Introduction

The transformation of healthcare systems worldwide has been shaped by the rise in neoliberal economic policies since the late 20th century [1]. With the shift away from Keynesian welfare state models, privatization has become a dominant mechanism in the restructuring of public services [2]. Although privatization is often defended on the grounds of improving efficiency and service quality, debates persist regarding its effects on accessibility, equity, and healthcare services. Unlike industrial privatization, privatization in the healthcare sector does not always entail a direct transfer of ownership; instead, it is carried out through various means, such as resource allocation, regulatory changes, and the expansion of private service providers integrated into public financing [3,4]. Chakravarty et al. [5] reported that for-profit hospitals were the quickest to enter expanding markets. The global private hospital market alone was valued at USD 712.6 billion in 2018 and is projected to reach nearly USD 2 trillion (USD 1967.6 billion) by 2027, with an annual growth rate of 11.9% [6]. The size of the private hospital sector varies depending on country-specific regulatory frameworks and healthcare infrastructure. Nordic countries with robust public sector infrastructure demonstrate a low demand for private hospitals, whereas countries such as India have a high demand for such [7,8].

The restructuring of the hospital sector in Turkey has been a key component of comprehensive healthcare reforms initiated in the early 2000s [9]. The Health Transformation Program (HTP), implemented in 2003, introduced significant regulatory and financial changes that redefined the relationship between public and private healthcare providers. While public hospitals maintained their dominance in critical care services, private hospitals rapidly expanded, gaining greater access to public insurance funds, which facilitated their growth. Additionally, a 2005 regulation allowed private hospitals to sign contracts with the Social Security Institution (SGK), enabling them to accept insured patients [10,11]. This transformation did not lead to the direct privatization of public hospitals; however, it resulted in the commercialization of hospital services, shifting the balance between public and private healthcare providers [9]. In 2008, the introduction of the General Health Insurance system brought major changes to the financing of healthcare services. Private hospitals gained broader access to SGK payments, contributing to the growth of the private sector. A portion of the demand for public hospitals was redirected toward private hospitals, leading to a rapid increase in the proportional share of private hospitals [12]. In 2010, the Full-Time Law was enacted, restricting doctors working in public hospitals from practicing in private hospitals. As a result, private hospitals increased their investments to strengthen their medical staff. Shortly after, in 2011, the city hospital model was introduced [13]. However, during this transition, gaps emerged due to the closure of existing public hospitals and the delay in opening new ones [14]. Private hospitals stepped in during this period, increasing their service capacity and attracting more patients. The Ministry of Health’s decision to further centralize public hospitals provided a more flexible service model for private hospitals, giving them a competitive advantage. Additionally, as the Full-Time Law came into effect, some doctors left public hospitals and transitioned to the private sector, further contributing to the expansion of private hospitals [12,13,14].

The privatization of healthcare services has often been justified as a mechanism to reduce waiting times and improve efficiency. However, different privatization models yield varying effects on accessibility and social welfare. Guan Xu et al. [15] analyzed two different privatization reforms—competition and collaboration—and found that their impact depends on government reimbursement levels and the degree of privatization in public–private partnerships. When government support is high but privatization levels are low, competition-driven privatization can improve service quality through market mechanisms. In contrast, in systems with higher privatization levels but moderate reimbursement rates, collaborative models between public and private hospitals may be more suitable in terms of patient welfare and social equity [15,16]. Similarly, Kowalik (2023), in analyzing the financial performance of hospitals in Poland after their transition from public ownership to commercial entities, found improvements in profitability but emphasized that service quality and equity effects must also be considered [17]. The Turkish case reflects elements of both approaches: private hospitals compete within a system partially integrated with public insurance schemes, while public hospitals continue to dominate in critical care services. This hybrid structure raises important questions about efficiency, financial sustainability, and equitable healthcare access, particularly for disadvantaged populations [16]. In private entrepreneurship, the profit maximization motive takes precedence over public health objectives. However, governments and public administrations influenced by neoliberalism have not refrained from implementing healthcare privatization policies. The historical consensus that healthcare services are a public service and essential for a strong state, which emerged with the modern nation-state, appears to have been disrupted. Even in the United Kingdom, where major political parties agreed that the National Health Service (NHS) should remain a public service, the system has gradually shifted toward privatization, particularly since the Thatcher era [18]. The initial stage of privatization involved reducing state investments in healthcare and public health expenditures. The expanding private sector, which replaced the public sector, quickly commercialized healthcare services, aligning them with market operating principles [19].

This study examines privatization trends in the hospital sector in Turkey between 2002 and 2022 through three key dimensions: physical capacity, service utilization, and workforce distribution. Using long-term data from the Ministry of Health, the study analyzes proportional trends between the public and private sectors regarding hospital bed numbers, service volumes (outpatient visits, inpatient admissions, and surgical procedures), and healthcare workforce employment. Breakpoint analysis identifies the impact of policy changes in 2005, 2008, and 2011, highlighting critical transformation moments that promoted the growth of the private sector while restricting the expansion of the public sector. By linking these breakpoints to specific policy changes, the study reveals that the privatization process in the healthcare sector cannot be explained by a simple public–private dichotomy. While emphasizing how structural changes in the hospital sector align with broader neoliberal trends, the study also highlights the tensions between market mechanisms and public health priorities. The findings provide insights into the growing role of private healthcare providers in Turkey and contribute to policy discussions on how healthcare services should be managed in terms of efficiency, accessibility, and equity.

## 2. Materials and Methods

### 2.1. Focus of the Study

The Turkish healthcare sector has undergone considerable transformations over the past two decades. This study aimed to analyze the changes in the hospital sector (public and private) during the period of 2002–2022, identifying key structural breakpoints and evaluating these changes in line with the objectives of health policies. The hospital sector was examined under three main categories: physical capacity, service utilization, and key parameters of the healthcare workforce. A comparative analysis on the distribution and trend differences between the public and private sectors was conducted.

### 2.2. Research Design

To address the research objectives and evaluate the structural shifts in Türkiye’s hospital sector between 2002 and 2022, a comprehensive methodological approach integrating trend analysis, structural breakpoint analysis, and segmented regression was employed. The study focuses on three key dimensions: physical capacity, service utilization, and healthcare workforce, allowing for a detailed comparison of public and private hospital trends.

Given the long-term nature of the dataset and the structural shifts in policy implementations over time, Mann–Kendall trend analysis was chosen as a non-parametric method to detect monotonic trends, while breakpoint analysis was applied to identify structural shifts within the dataset. The findings from these methods were further refined using segmented regression models to evaluate how trends evolved before and after breakpoints.

### 2.3. Type of Research and Data Used

The data (Supplementary File: Healthcare Services Indicators) used in this study were extracted from 15 comprehensive health statistics yearbooks published by the Ministry of Health [20]. This retrospective longitudinal study used secondary data obtained from statistical reports issued by the Ministry of Health. University hospitals were excluded from the analysis for two reasons:

(1) In the Ministry of Health datasets, data on university hospitals included public universities and foundation hospitals categorized as private hospitals, making them unsuitable for a clear public–private comparison.
(2) University hospitals, mainly established for educational purposes, have unique developmental characteristics that do not align with the objectives of this analysis.

The analysis focused on key indicators of healthcare capacity, service utilization, and healthcare workforce over a 21-year period (2002–2022). A comparative assessment was conducted to evaluate the growth, distribution, and trend differences between public and private hospital sectors, providing insights into the privatization process based on these metrics. University hospitals were excluded from the analysis due to classification inconsistencies in the Ministry of Health datasets. These hospitals are categorized under both public (state universities) and private (foundation universities), making direct comparisons challenging. Additionally, foundation hospitals, which are technically private entities, further complicate the classification. To maintain consistency in the comparison, university hospitals were omitted from the dataset. Consequently, the share of hospitals not classified under either public or private hospitals in the reported statistics represents university hospitals. The analysis, therefore, focused solely on the following parameters for public and private hospitals:

- **Physical Capacity:**
  ○ *Ratio of hospitals:* The proportion of hospitals within the public and private sector relative to the total number of hospitals in the system.
  ○ *Ratio of hospital beds:* The percentage share of public and private sector hospital beds in relation to the total hospital bed capacity.
  ○ *Ratio of qualified beds:* The proportion of beds meeting regulatory qualifications (e.g., intensive care, specialized units) within public and private hospitals.
  ○ *Ratio of intensive care beds:* The percentage of total intensive care unit beds allocated within public and private hospitals.
  ○ *Ratio of the dialysis devices:* The total number of dialysis machines divided by the number of hospitals within each sector, reflecting the availability of dialysis services per institution.
- **Service Utilization:**
  ○ *Ratio of outpatient visits:* The proportion of outpatient visits handled by public and private hospitals relative to the total number of outpatient visits.
  ○ *Ratio of inpatient cases:* The share of inpatient admissions in public and private hospitals.
  ○ *Ratio of surgeries:* The proportion of total surgical procedures performed in public and private hospitals
  ○ *Ratio of hospital days:* The distribution of total hospitalization days spent in public versus private hospitals.
- **Healthcare Workforce:**
  ○ *Ratio of doctors:* The proportion of employed doctors within public and private hospitals.
  ○ *Ratio of nurses and midwives:* The share of nurses and midwives working in public versus private hospitals.

### 2.4. Research Hypotheses

The study was based on the following hypotheses:

**H_1_.**  *Significant differences exist in the physical capacity indicators for healthcare services between the public and private hospital sectors during the period of 2002–2022.*

**H_2_.**  *Significant differences exist in service utilization trends over time between the public and private hospital sectors.*

**H_3_.**  *Significant changes have occurred in the employment of the healthcare workforce (doctors, nurses, and midwives) in the public and private hospital sectors.*

**H_4_.**  *Overall trend differences between the public and private hospital sectors are statistically significant.*

### 2.5. Data Analysis

Mann–Kendall trend analysis was applied to assess long-term monotonic trends in the hospital sector. This test is particularly suitable for datasets spanning multiple decades as it is non-parametric, making it robust to non-normality and outliers that could otherwise distort findings in parametric trend analyses [21,22]. To account for policy shifts and structural changes, breakpoint analysis was used to identify points in time where the trends exhibited significant shifts. This approach allows for detecting critical periods of reform, regulatory intervention, or economic shocks that impacted the hospital sector. Since Mann–Kendall analysis identifies the presence of trends but does not provide insight into how trends evolve before and after breakpoints, segmented regression models (broken-stick regression) were incorporated [21]. To determine the optimal number of breakpoints in the segmented regression models, Bayesian information criterion (BIC) and residual sum of squares (RSS) were employed. The BIC was selected because it accounts for model complexity and penalizes overfitting, ensuring that the final model balances explanatory power with parsimony. The RSS, on the other hand, measures the total deviation of the data from the model’s predicted values, helping to evaluate how well the segmented regression fits the observed trends. BIC was preferred for determining the final breakpoint models because it prioritizes models that generalize well without overfitting, ensuring that the identified breakpoints reflect meaningful structural changes rather than noise. This method estimates the slopes before and after breakpoints, enabling a refined assessment of whether trends accelerated, decelerated, or remained stable post-reform periods [23,24].

Data were analyzed using the R software version 4.3.1 with the strucchange, trend, and ggplot2 packages [25,26,27]. Trend analysis was conducted using the Mann–Kendall trend test, a non-parametric method independent of distribution assumptions, which tests for monotonic trends in time series data [28]:

$$S=\sum_{i=1}^{n-1}\sum_{j=i+1}^{n}sgn(x_{j}-x_{i})$$

$$sgn\left(x\right)=\left\{\begin{array}{cc} 1 & if\;x>0\\ 0 & if\;x=0\\ -1 & if\;x<0 \end{array}\right.$$

$$Z=\left\{\begin{array}{cc} \frac{S-1}{\sqrt{Var(S)}} & if\;S>0\\ 0 & if\;S=0\\ \frac{S+1}{\sqrt{Var(S)}} & if\;S<0 \end{array}\right.$$

The trends for each variable were calculated. Physical capacity, service utilization, and healthcare workforce were measured as proportional averages. An overall proportional average for all variables was also included in the analysis. The structural breakpoints in the trends were identified via breakpoint analysis, which detects whether relationships between dependent and independent variables structurally change over time [29]:

$$y_{t}=\alpha_{j}+\beta_{j}t+\epsilon_{t},\;for\;t<t\leq t_{j+1},\;j=1,\;\ldots ,\;k$$

The results were validated using the Bayesian information criterion (BIC) and residual sum of squares (RSS) values [30,31]:

$$BIC=kln\left(n\right)-2ln(\hat{L})$$

$$RSS=\sum_{i=1}^{n}{\left(y_{j}-{\hat{y}}_{i}\right)}^{2}$$

The trend differences between the public and private sectors were assessed using the Z-difference test [32]:

$$Z_{diff}=\frac{\left|Z_{public}-Z_{private}\right|}{\sqrt{2}}$$

The statistical significance of the trends was expressed using *p*-values, and the strength of the trends (ranging from −1 to +1) was determined using Kendall’s Tau coefficient [33]. To analyze the trend changes based on the structural breakpoints, the segmented regression and broken-stick models were applied. The broken-stick regression detects how trends change post-breakpoints, determining whether trends increase, decrease, or stabilize [34]:

$$y_{t}=\left\{\begin{array}{c} \begin{array}{cc} \alpha_{1}+\beta_{1}t, & if\;t\leq \;t_{1}\\ \alpha_{2}+\beta_{2}t & if\;t_{1}<t\leq \;t_{2}\\ \alpha_{3}+\beta_{3}t & if\;t_{2}<t\leq \;t_{3} \end{array}\\ \begin{array}{cc} \vdots & \vdots \\ \alpha_{k}+\beta_{k}t & if\;t_{k-1}<t\leq \;t_{k} \end{array} \end{array}\right.$$

The Mann–Kendall test assumes independence and is sensitive to autocorrelation; we conducted diagnostic tests and applied pre-whitening techniques where necessary [22]. Additionally, as the segmented regression model relies on linearity within segments and homoskedasticity, we tested for heteroskedasticity using the Breusch–Pagan test and applied generalized least squares (GLS) corrections where required. To mitigate autocorrelation, the Durbin–Watson test was performed, and adjustments such as the Cochran–Orcutt transformation and Newey–West estimators were applied when needed. Recognizing the impact of missing values on both methods, we ensured data completeness by cross-validating hospital statistics from multiple Ministry of Health reports, while minor gaps were addressed using interpolation techniques without introducing artificial trends. These refinements enhance the robustness and validity of our findings, ensuring that data quality concerns do not compromise the integrity of the results. All findings were evaluated at a 95% confidence level.

## 3. Results

Table 1 presents the percentage distributions of various health service indicators between the public and private sectors from 2002 to 2022. The indicators included hospital beds, qualified hospital beds, intensive care beds, dialysis centers, dialysis machines, outpatient visits, inpatient numbers, surgeries, hospitalization days, physicians, and nurses/midwives.

In 2002, the public sector held 66.96% of the services, whereas the private sector accounted for 23.44%. By 2022, the share of the public sector slightly decreased to 58.84%, whereas that of the private sector increased to 36.78%. In 2002, the public sector accounted for 65.30% of the total hospital beds, while the private sector held a 7.53% share. By 2022, the private sector’s share increased to 21.00%, whereas the public sector’s share experienced a slight decline to 62.25%, reflecting a 3.05 percentage point reduction. As regards the qualified hospital beds, the public sector had a 36.12% share in 2002, which increased to 64.10% in 2022. Meanwhile, the share of the private sector dropped from 30.07% to 20.91% over the same period. In terms of intensive care beds, the private sector had a higher share in 2002 at 44.81% compared with the public sector, which had 39.25%. However, by 2022, the share of the public sector increased to 49.46%, whereas that of the private sector decreased to 36.15%. In 2002, the shares of the private and public sectors for dialysis machines were 51.30% and 30.87%, respectively. By 2022, these shares increased to 56.44% and 37.06%, respectively. In terms of outpatient visits, the share of the public sector was 88.32% in 2002, which decreased to 75.72% in 2022, whereas that of the private sector increased from 4.58% to 15.07%. For inpatients, the share of the public sector fell from 75.70% in 2002 to 53.97% in 2022, whereas that of the private sector increased from 10.10% to 30.63%. A similar trend was observed for surgeries, with the share of the public sector decreasing from 67.09% in 2002 to 51.92% in 2022, whereas that of the private sector increasing from 13.69% to 29.42%. As regards hospitalization days, the share of the public sector decreased from 73.79% to 60.12%, whereas that of the private sector increased from 5.37% to 20.61%. Finally, in 2002, the public sector employed 62.40% of physicians and 82.40% of nurses/midwives. By 2022, the share of physicians in the public sector had declined to 60.10%, while the share of nurses/midwives had decreased to 74.40%. In contrast, the private sector increased its share of physicians from 15.70% to 18.30% and its share of nurses/midwives from 9.70% to 13.10% (Table 1).

**Figure 1:**
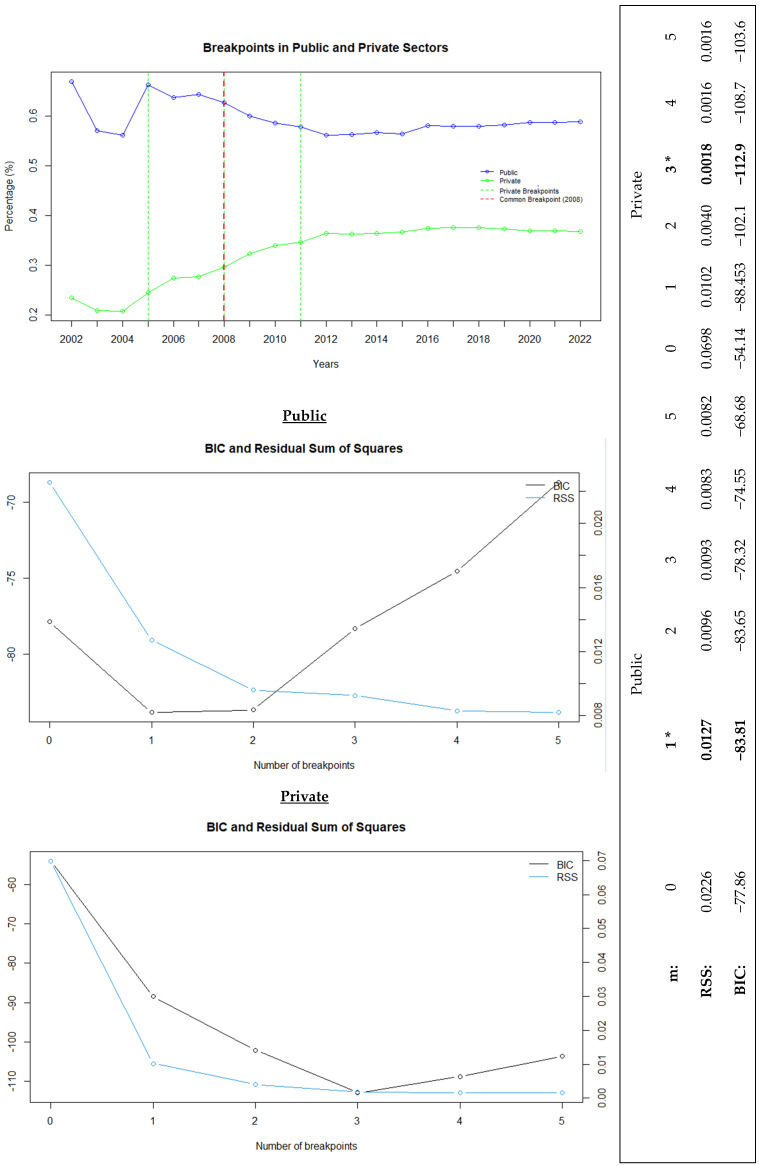
Public and private healthcare trends with breakpoint analysis and model fit comparison. “*” represents the best recommended breakpoint.

Figure 1 presents the multivariate analysis of the common trends and average changes over time for all variables modeled in physical capacity, healthcare workforce, and service utilization in the public and private hospital sectors. It also highlights the breakpoints (m) and model fit. The model fit was achieved by selecting the optimal number of breakpoints using the BIC, which penalizes model complexity. A lower BIC value indicates a better model fit.

The error margin of the model was assessed using the RSS, which was calculated by summing the squared differences between the predicted and actual values. Then, the model fit of the data with the number of breakpoints determined using the BIC was evaluated. Based on this analysis, the single-breakpoint model for the public sector (BIC: −83.81) and the three-breakpoint model for the private sector (BIC: −112.9) yielded the best results, as confirmed by the acceptance of the RSS values.

In the breakpoint analysis conducted to identify sudden changes over time, 2008 was determined as the breakpoint year for the public sector. For private hospitals, the first breakpoint occurred in 2005, marking the start of an upward trend. This trend accelerated with the second breakpoint in 2008 and continued until 2011, after which a slower growth was observed. Thus, these 3 years were identified as the breakpoints for private hospitals. Ultimately, the year 2008 emerged as a common breakpoint for public and private hospitals (Figure 1).

### 3.1. Physical Capacity

Under the category of physical capacity, the infrastructure used in the delivery of healthcare services was analyzed through two components: basic health infrastructure and advanced health infrastructure.

#### 3.1.1. Basic Physical Health Infrastructure

**Figure 2:**
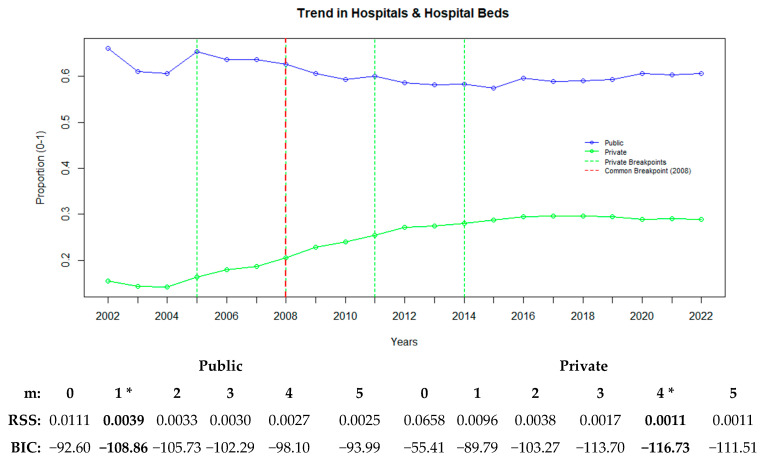
Public and private sector trends in hospitals and hospital beds with breakpoints. “*” represents the best recommended breakpoint.

Basic physical health infrastructure refers to the essential elements required for the provision of healthcare services. In this context, the ratios of the number of hospitals and the ratios of hospital beds were used. Figure 2 illustrates how the changes in the basic physical health infrastructure of the public and private sectors occurred over time and shows the years in which the breakpoints were observed.

Figure 2 illustrates the common trends and breakpoints for the variables related to hospitals and hospital beds in the public and private sectors. The average changes in the ratios of the numbers of hospitals and hospital beds indicate significant shifts in the basic physical health infrastructure.

For the public sector, a single-breakpoint model was determined to be appropriate (BIC: −108.86), with the breakpoint identified in 2008. In the private sector, a four-breakpoint model was found to be suitable (BIC: −116.73), with the breakpoints occurring in 2005, 2008, 2011, and 2014.

In the public sector, the decreasing trend stabilized after the 2008 breakpoint. In contrast, in the private sector, the 2005 breakpoint marked an acceleration in growth, which became more pronounced with the 2008 breakpoint. The growth rate slowed down in 2011, and the trend leveled off after the 2014 breakpoint (Figure 2).

#### 3.1.2. Advanced Physical Health Infrastructure

**Figure 3:**
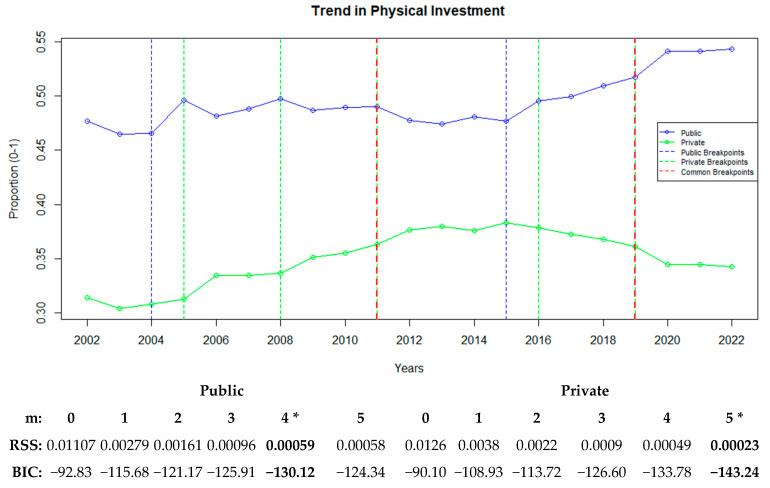
Public and private sector trends in physical investment with breakpoints. “*” represents the best recommended breakpoint.

Under advanced physical health infrastructure, additional variables indicative of the infrastructure required for the provision of higher-level and higher-quality healthcare services were included in the analysis. In this context, along with the ratios of the number of hospitals and hospital beds, variables such as the ratios of qualified hospital beds, intensive care beds, and dialysis devices were incorporated for the public and private sectors. The temporal changes in the averages of all variables examined as part of physical capacity were analyzed. Figure 3 depicts the trends in physical capacity and identifies the years in which the breakpoints occurred.

Figure 3 highlights the trends and breakpoints in the physical capacity variables for the public and private hospital sectors. The analysis revealed considerable changes in physical capacity, including variables such as the numbers of hospitals, hospital beds, qualified hospital beds, intensive care beds, and dialysis devices.

For the public sector, four breakpoints were identified in 2004, 2011, 2015, and 2019, corresponding to the critical changes in the physical investment ratios during these years. In the private sector, five breakpoints were identified in 2005, 2008, 2011, 2016, and 2019. The first major increase in the private sector began in 2005, which accelerated in 2008 and remained steady until 2016. The years 2011 and 2019 emerged as common breakpoints for both sectors, but with contrasting trends. The lowest BIC value for the public sector was observed in the four-breakpoint model (−130.12), whereas the five-breakpoint model exhibited the best fit for the private sector (−143.24). When examining the breakpoints for each variable, a single structural breakpoint for the ratio of hospitals in the public sector occurred in 2008, after which a continuous downward trend was observed. In the private sector, the breakpoints for the ratio of hospitals occurred in 2005, 2008, and 2011, which were all associated with a continuous upward trend. These breakpoints reflect a stabilizing trend in the public sector, whereas in the private sector, the growth slowed down and eventually plateaued (Figure 3).

### 3.2. Healthcare Service Utilization

**Figure 4:**
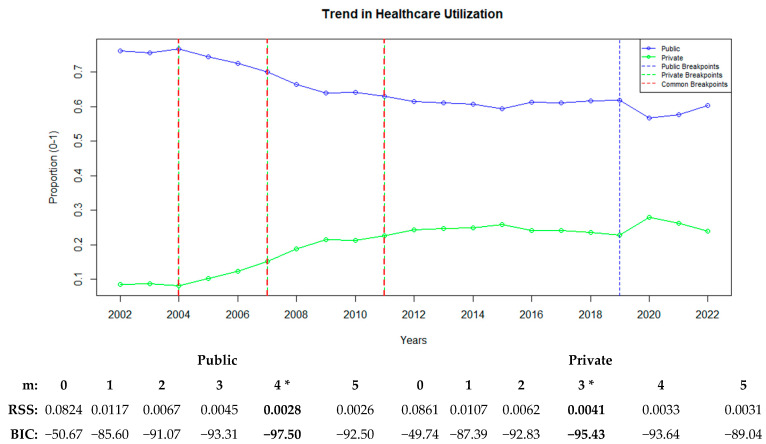
Public and private sector trends in healthcare utilization with breakpoints. “*” represents the best recommended breakpoint.

Figure 4 illustrates how healthcare service utilization has changed over time and shows the breakpoints in the public and private hospital sectors. The analysis revealed changes in the utilization ratios of key hospital services, such as outpatient visits, inpatient cases, hospital days, and surgeries. Based on the BIC and RSS values, a four-breakpoint model was found to best fit the public sector (BIC: −97.50), whereas a three-breakpoint model was optimal for the private sector (BIC: −95.43).

The public sector experienced its first breakpoint in 2004, followed by additional breakpoints in 2007, 2011, and 2019, demonstrating a generally decreasing trend in service utilization. In the private sector, three breakpoints were identified in 2004, 2007, and 2011. Starting with the shared breakpoint in 2004, public healthcare utilization exhibited a consistently declining trend, whereas the private sector showed an increasing trend. Another shared breakpoint in 2007 exhibited intensified trends, with acceleration in increases and decreases in the private and public sectors, respectively. In 2011, a breakpoint signaling the stabilization of these trends occurred for both sectors. In addition, the public sector experienced a breakpoint in 2019, which led to a renewed acceleration in the declining trend (Figure 4).

### 3.3. Healthcare Workforce

**Figure 5:**
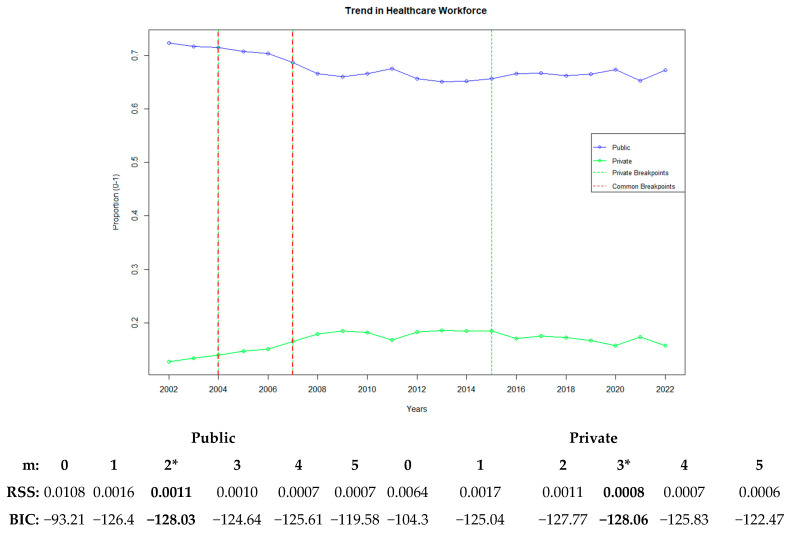
Public and private sector trends in the healthcare workforce with breakpoints. “*” represents the best recommended breakpoint.

Figure 5 illustrates how the healthcare workforce in the public and private hospital sectors has changed over time and shows the years in which breakpoints occurred.

Figure 5 illustrates the trends and breakpoints related to the healthcare workforce. In this context, changes in the ratios of doctors and nurses/midwives in the public and private sector were evaluated. In the public sector, the breakpoints occurred in 2004 and 2007, with the optimal model determined using the BIC (−128.03). The 2004 breakpoint showed a declining trend whereas the 2007 breakpoint exhibited stabilization in the distribution of the healthcare workforce, including doctors and nurses/midwives. In the private sector, the breakpoints occurred in 2004, 2007, and 2015, with the optimal model determined using the BIC (−128.06). The 2004 breakpoint displayed an increasing trend, the 2007 breakpoint showed stabilization, and the 2015 breakpoint marked the beginning of a declining trend.

Up to this section, sector-based trends in hospital infrastructure, service utilization, and workforce distribution have been examined, and critical breakpoints indicating shifts in healthcare policies and privatization dynamics have been identified. Following this section, the Mann–Kendall trend test was applied to analyze different trend patterns and sectoral transformations, and segmented regression was used to assess the significance of breakpoints over time.

### 3.4. Comparison of the Public and Private Sector Trends

The results of the Mann–Kendall trend test conducted for the variables examined in the public and private hospital sectors as well as the evaluation of public–private sector trend differences using the Z-difference test are presented in Table 2.

For each variable, the table provides *p*-values, z-statistics, and Kendall Tau coefficients for the public and private sectors. In addition, the Z-difference values representing the trend differences between the two sectors and the corresponding *p*-values are included.

The overall trend based on the averages of all variables in the public sector was statistically insignificant, negative, and weak (τ = −0.258, *p* = 0.104). In contrast, the overall trend in the private sector was positive, moderate, and statistically significant (τ = 0.373, *p* = 0.019). According to the Z-difference test results, the difference in the general trends between the public and private sectors was significant (Z-Diff = 4.516, *p* = 1.33 × 10^−15^), indicating decreasing and increasing trends in the public and private sectors, respectively.

For the average ratio of the number of hospitals and hospital beds, the public sector exhibited a negative, moderate, and statistically significant trend (τ = −0.400, *p* = 0.0122), whereas the private sector showed a positive, highly significant trend with excellent strength (τ = 0.809, *p* = 3.338 × 10^−7^). The Z-difference test confirmed a significant difference between the sectors, with decreasing and increasing trends in the public and private sectors, respectively (Z-Diff = 5.380, *p* = 7.41 × 10^−8^).

In the physical capacity dimension, which includes all physical investment variables, the public sector demonstrated a strong, positive, and significant trend (τ = 0.562, *p* = 2.04 × 10^−4^). The private sector also displayed a positive, moderate, and significant trend (τ = 0.41, *p* = 1.026 × 10^−2^). However, the trend difference between the two sectors was not statistically significant (*p* > 0.05). Regarding healthcare utilization, the public sector showed a strong, negative, and significant trend (τ = −0.752, *p* = 8.56 × 10^−7^), whereas the private sector demonstrated a strong, positive, and significant trend (τ = 0.676, *p* = 2.06 × 10^−5^). The Z-difference test indicated a highly significant difference between the sectors (Z-Diff = 6.46, *p* = 1.98 × 10^−10^), confirming decreasing and increasing trends in the public and private sectors, respectively. For the healthcare workforce, the public sector exhibited a moderate, negative, and significant trend (τ = −0.465, *p* = 3.69 × 10^−3^), whereas the private sector showed a positive but not statistically significant trend (τ = 0.278, *p* = 8.49 × 10^−2^). The Z-difference test indicated a significant difference between the sectors (Z-Diff = 3.27, *p* = 1.07 × 10^−3^), with a decreasing trend in the public sector and no significant increase in the private sector (Table 2).

The Mann–Kendall trend analysis has identified the direction and statistical significance of trends in the public and private hospital sectors. However, this test does not measure the exact time points of trend changes or variations in slopes. Therefore, segmented regression was applied to identify significant structural breakpoints and evaluate trend changes.

### 3.5. Evaluating Slopes After Breakpoints with Segmented Regression

After conducting the Mann–Kendall trend test and a comparative analysis of trends in the public and private hospital sectors, this section employs segmented regression to examine structural changes in the healthcare sector in greater detail. While the previous analyses identified overall trends and significant differences between sectors, segmented regression provides a more detailed assessment by detecting changes in slope before and after breakpoints. Additionally, this section determines whether these breakpoints are statistically significant. Estimating the slopes before and after each breakpoint helps to understand the responses to major policy changes, economic fluctuations, and sectoral transformations.

In the analysis, five key dimensions—physical capacity, healthcare utilization, human resources, hospital infrastructure, and overall sectoral performance—were evaluated using broken-stick regression models. The statistical assumptions of segmented regression, including normality, homoskedasticity (variance homogeneity), and autocorrelation, were tested before the analysis, and necessary corrections were applied where required (see Supplementary File). When needed, estimations were enhanced using Newey–West or Cochran–Orcutt estimators to ensure robust results. The results of the analyses are presented in Table 3.

The results in Table 3 and the general trend, which includes all variables, were analyzed, and the year 2008 was identified as the breakpoint for the public sector. At this point, the annual increase (−0.002572) was statistically significant (*p* < 0.0006). The fluctuating trend in the public sector experienced a breakpoint in 2008, after which the decline rate slowed down (β = 0.0103504; *p* = 1.39 × 10^−8^).

In the private sector, the breakpoints occurred in 2005, 2008, and 2011 (U1, U2, and U3, respectively), with values of 0.0164, −0.0083, and −0.010, respectively. These values were statistically significant (*p* < 0.0002, *p* < 0.0021, *p* < 0.00023). After 2005, an increasing trend in healthcare utilization was observed in the private sector, but it slowed down and stabilized in 2008 and 2012, respectively. Overall, the share of the public sector decreased over time but stabilized in recent years, whereas that of the private sector exhibited growth, which also stabilized in recent years.

In the model that included the ratio of hospitals and hospital beds, 2008 was identified as the breakpoint for the public sector. At this point, the annual decrease (0.6496) was not significant (*p* = 0.6496). After the 2008 breakpoint, the fluctuating trend in the public sector slowly increased (β = 0.009080; *p* = 0.00187).

In the private sector, the breakpoints occurred in 2008, 2011, 2015, and 2019 (U1, U2, U3, and U4, respectively), with values of 0.0249, 0.0058, −0.0105, and −0.0073, respectively. Except for U2, these values were statistically significant. The general trend in the private sector exhibited a significant increase (β = 0.0086171; *p* = 0.01317). Furthermore, a significant acceleration in growth occurred in 2008 (β = 0.024939; *p* = 0.0111). The growth insignificantly continued at the 2011 breakpoint (β = 0.005851; *p* = 0.3379) but significantly slowed down in 2015 (β = −0.010535; *p* = 2.30 × 10^−5^) and stabilized with a slower rate of decline in 2019 (β = −0.007374; *p* = 0.00198).

For the physical capacity, the general trend in the public sector after the 2004 breakpoint fluctuated and was statistically insignificant (β = 0.015931). In 2011, it significantly shifted to a decline (β = −0.009180, *p* = 0.0296 *), but in 2015, it returned to a significant increase (β = 0.014515, *p* = 5.90 × 10^−5^ *). In 2019, the increasing trend stopped (β = −0.009019), but this change was not statistically significant (*p* = 0.4236).

In the private sector, a dramatic increase was observed in 2005 (β = 0.020309), followed by declines in 2008 and 2011. A significant change occurred after the 2016 breakpoint, which transitioned to a declining trend (β = −0.010437, *p* = 0.0184). In 2019, the declining trend slowed down, but the change was not statistically significant (β = 0.0184; *p* = 0.4193).

For healthcare utilization, the public sector exhibited a declining trend starting with the 2004 breakpoint (β = −0.025300). This decline intensified with the 2011 breakpoint (β = −0.030030) and began stabilizing with a reduced decline rate in 2011 (β = 0.023520) and 2019 (β = 0.025960). All post-breakpoint slopes were statistically significant (*p* < 0.05).

In the private sector, the 2004 breakpoint exhibited an increasing trend (β = 0.029997). But in 2008, a statistically significant slowdown in this trend occurred (β = −0.01806, *p* = 0.0475). By 2011, the slowdown became more pronounced (β = −0.009753), although it was not statistically significant (*p* = 0.1264).

For the healthcare workforce, the public sector experienced a declining trend with the 2004 breakpoint (β = −0.016000), followed by a stabilization in 2007 (β = 0.021366). However, these changes in the slopes were not statistically significant (*p* > 0.05). In the private sector, an increasing trend followed the 2004 breakpoint (β = 0.007557), which later stabilized (β = −0.012800) with a statistically significant result (*p* = 0.021). The 2011 breakpoint marked the onset of a declining trend, although it was not statistically significant (*p* = 0.106) (Table 3).

## 4. Discussion

The Turkish healthcare sector underwent considerable transformations during the period of 2002–2022. The findings of this study indicated notable differences in the development of physical capacity, service utilization, and healthcare workforce between the public and private hospital sectors. Furthermore, these findings provide an important foundation for evaluating the impact of health policies on the balance between the public and private sectors and discussing the causes of structural breakpoints in the delivery of healthcare services.

The declining trend in the public hospital sector and the increasing trend in the private hospital sector indicate a restructuring of public–private dynamics in Turkey’s healthcare system. The common structural breakpoints observed in 2008 and 2011 highlight the impact of healthcare policies during these periods. The Regulation on Private Healthcare Institutions Providing Outpatient Diagnosis and Treatment, published in the Official Gazette on February 15, 2008, halted new private hospital pre-authorization applications and requests from existing private hospitals to expand their medical service units, healthcare personnel, or high-tech medical equipment, thereby increasing the value of private hospital licenses and creating a market where existing licenses were actively traded [35]. The rapid expansion of the private hospital sector initially led to a shortage of specialist physicians in the public sector, prompting the establishment of the Planning and Employment Commission within the Ministry of Health. This commission was formally established by a regulation published in the Official Gazette on 11 March 2009 (No. 27166) and was implemented as a policy intervention to ensure the balanced allocation of healthcare resources and address workforce disparities between the public and private sectors [36]. Our findings demonstrate that the structural breakpoints identified in 2008 and 2011 align directly with these policy changes. While the public sector has gained a more stable structure in service delivery, the private sector expanded after certain breakpoints and eventually reached a more stabilized state. This transformation in healthcare services underscores the necessity of sustainable workforce management and strategic adjustments to maintain the balance between the public and private sectors [35,36,37].

The findings on structural breakpoints in public hospitals indicate significant changes in 2011, 2015, and 2019. The notable decline in physical capacity in 2011 (β = −0.009180, *p* = 0.0296) can be associated with the transition to the city hospital model as part of the Health Transformation Program. During this period, some public hospitals were closed or merged, but since new investments had not yet been completed, the total bed capacity of public hospitals declined [38]. By 2015, a recovery in the public sector was observed (β = 0.014515, *p* = 5.90 × 10^−5^), which can be explained by the addition of new hospital beds with the opening of city hospitals. Our findings on healthcare utilization show that demand for public hospitals began to decline in 2004 (β = −0.025300, *p* < 0.05) and this decline accelerated in 2011 (β = −0.030030, *p* < 0.05). This trend can be attributed to the Health Transformation Program launched in 2003, which enabled private hospitals to sign agreements with the Social Security Institution and receive reimbursements from public insurance. This process led to a shift in patients toward the private sector, reducing the utilization rates of public hospitals. Additionally, with the implementation of the Full-Time Law in 2011, many doctors in public hospitals transitioned to private hospitals, further reducing public healthcare capacity [39]. However, after 2011, the rate of decline in public hospital utilization slowed down (β = 0.023520, *p* < 0.05), and by 2019, stabilization was achieved in the public sector (β = 0.025960, *p* < 0.05). This trend can be explained by the completion of city hospitals, which increased capacity and strengthened public hospitals. The decline in the physical capacity of public hospitals and the reduction in healthcare utilization appear to be largely driven not only by the expansion of private hospitals but also by structural transformations within the public healthcare system, including the closure or merging of some public hospitals during the transition to the city hospital model, the enactment of the Full-Time Law in 2011 leading to the transfer of public hospital doctors to the private sector, and the implementation of the Public Hospital Unions system in 2012, which altered the management structure of public hospitals, led to the introduction of the General Health Insurance system in 2008, which allowed private hospitals to operate within the public insurance framework, and the outsourcing of certain public hospital services to the private sector. Considering the timing of the structural breakpoints, these findings align with the conclusion that these transformations are closely linked to the observed shifts in the public healthcare system [13,14,39].

For the private hospital sector, 2008, 2015, and 2019 were identified as key structural breakpoints. Our findings indicate that in 2008, there was a significant acceleration in the growth rate of the private hospital sector (β = 0.024939, *p* = 0.0111). This surge can be attributed to the implementation of the General Health Insurance system, which allowed private hospitals to receive greater reimbursements from the Social Security Institution [38,40]. This policy provided a substantial financial incentive for private hospitals, boosting investment in the sector and accelerating the establishment of new hospitals. However, by 2015, the growth rate of the private hospital sector had significantly declined (β = −0.010535, *p* = 2.30 × 10^−5^). This trend may be linked to changes in Social Security Institution reimbursement policies and an increase in patient co-payments at private hospitals. The government’s efforts to control healthcare expenditures directed toward private hospitals may have led to a decrease in patient preference for private healthcare services and a slowdown in the sector’s growth rate [40].

By 2019, the growth of private hospitals had largely stabilized, with a lower decline rate leading to equilibrium (β = −0.007374, *p* = 0.00198). This trend can be explained by the sector reaching saturation, increased competition, and the resurgence of public hospitals, as observed in China, where regulatory reforms and mixed payment models influenced the balance between public and private hospital growth [41]. In terms of physical capacity, a declining trend in private hospitals began in 2016 (β = −0.010437, *p* = 0.0184). Our findings suggest that this decline may be linked to a reduction in incentives for private hospital investments and the strengthening of the public sector through the city hospital model. Regarding healthcare workforce, we observed that employment in private hospitals increased after 2004 (β = 0.007557, *p* = 0.021), but in later years, this growth halted and stabilized (β = −0.012800, *p* = 0.021). With the implementation of the Health Transformation Program, the number of doctors and healthcare personnel in private hospitals increased; however, as the sector reached saturation, new employment opportunities became more limited [42]. Overall, the growth of the private hospital sector was initially driven by the allocation of public funding to the private sector, but over time, changes in healthcare policies and the strengthening of public hospitals highlighted the need to regulate this expansion [43]. Additionally, market saturation played a role in balancing this growth, contributing to the sector’s stabilization [39,40,42,43].

On the other hand, the increase in the private sector share cannot be evaluated as completely negative. For example, it may be beneficial for the public to focus on certain special services. Among South Asian countries, India exhibited a dramatic decline in the number of public hospital beds. The share of public hospital beds dropped from 71.2% in 1973 to 39.0% in 1996, whereas that of private hospital beds increased from 28.8% to 61.0%. In contrast, Sri Lanka, which has better health indicators than the other South Asian countries, has maintained widespread and relatively stronger public healthcare services [44]. A similar trend can be observed in Turkey. The share of public hospital beds (excluding university hospitals) decreased from 65.3% in 2002 to 62.25% in 2022, whereas that of private hospital beds increased from 7.53% to 21.00%. However, unlike in India, the proportion of qualified beds in Turkey significantly increased during the same period from 36.12% to 64.10%. When evaluated together with the increase in the public share of intensive care beds (49.46%), it can be considered an indicator that the new investments and hospital renovations in the public hospital sector are quality-oriented. Millar et al. [45] support our findings by emphasizing the necessity for public health to focus on more specialized and less profitable healthcare services while also highlighting the importance of guiding population health. Their research further suggests that prioritizing health outcomes over mere service provision is essential for the sustainability of the healthcare system.

Indeed, while our findings indicate the increasing role of private hospitals in Turkey’s healthcare system, they also suggest that the redistribution of service areas between public and private hospitals has occurred as a strategic shift. Between 2002 and 2022, the 82.3% increase in the number of private hospitals and the rise in the share of private hospital beds from 7.53% to 21.00% demonstrate the expansion of the private sector in routine and elective care services. However, although the public sector’s share of total hospital beds declined from 65.30% to 62.25%, the increase in the proportion of qualified beds from 36.12% to 64.10% suggests that public hospitals have increasingly focused on high-complexity services such as intensive care and dialysis. This shift indicates that public hospitals have prioritized critical care services and strengthened their role in managing high-complexity cases. In particular, health policies implemented after the COVID-19 pandemic have supported a strategic orientation toward enhancing emergency and intensive care capacity in public hospitals [46]. Similar trends have been observed in other countries, where the public healthcare system’s focus on less profitable but critical healthcare services has been recognized as a key strategy for ensuring the sustainability of healthcare systems. For example, in the Netherlands, the Zorg In Ontwikkeling (Zio) program, an integrated care network aimed at transitioning hospitals toward primary healthcare services, has focused on improving health outcomes for diabetes patients [47,48]. Such programs enable public hospitals to concentrate on more complex and critical care services, while private hospitals expand their role in providing routine and elective care services [48].

According to the Turkish Statistical Institute, out-of-pocket healthcare expenditures increased by 97.2% from 112.018 billion TL in 2022 to 220.914 billion TL in 2023, while per capita health expenditures rose by 104.2%, from 7141 TL to 14,582 TL during the same period [49]. This sharp increase indicates that households, particularly low-income groups, face a significant financial burden in accessing healthcare services. Evidence from OECD countries suggests that prepaid financing models, such as taxation-based healthcare systems, help reduce the risk of catastrophic healthcare expenditures. Countries with higher income levels and stronger financial protection mechanisms experience lower rates of economic hardship due to healthcare costs, underscoring the importance of strengthening healthcare financing structures to ensure equitable access and reduce financial burdens on households [50].

Despite their growing share, private hospitals remain primarily concentrated in urban centers, exacerbating geographical inequalities in healthcare access. According to the analysis by Torun, Atan, and Ayanoğlu (2020), public hospital efficiency varies significantly across regions, with hospitals in major cities in Southeastern Anatolia demonstrating higher efficiency than those in Western Marmara and other western regions [51]. However, Akgış İlhan (2022) highlights that urban centers in western regions benefit from an abundance of healthcare services, while rural and eastern regions face significant inequalities in hospital access. Network analysis shows that in some eastern and southeastern regions, travel times to the nearest hospital exceed 30 min, significantly restricting access compared to urban areas. Additionally, the unequal distribution of healthcare professionals further exacerbates regional disparities, with some regions reporting a high patient-to-doctor ratio (783 patients per doctor), deepening healthcare inequalities [16].

In response to these access challenges, various models have been proposed to improve healthcare delivery in rural areas. Telehealth initiatives, mobile healthcare services, and digital health applications have emerged as potential solutions to bridge the healthcare gap between urban and rural populations. Studies indicate that mobile health units and telemedicine programs can overcome geographical and infrastructural barriers, improving healthcare delivery [52,53]. However, the effectiveness of these solutions depends on factors such as digital literacy, infrastructure investments, and policy support for rural health programs. Given these inequalities, the rising share of private healthcare services poses additional risks for rural populations. If the expansion of private hospitals—primarily concentrated in urban centers—continues uncontrolled, healthcare resources may be further diverted from rural areas, where access to essential services is already limited. This underscores the increasing need for targeted policy interventions to ensure the equitable distribution of healthcare services across all regions.

Between 2020 and 2021, the decline in public hospital service utilization was not solely due to privatization but was also significantly influenced by the COVID-19 pandemic. Public hospitals were at the forefront of pandemic management, prioritizing COVID-19 patients, which led to a substantial decrease in elective procedures, outpatient visits, and non-urgent hospitalizations [54]. The pandemic also placed immense pressure on healthcare personnel, with staff shortages and burnout particularly affecting public hospitals, further restricting service capacity. Meanwhile, private hospitals—depending on their level of integration into the pandemic response—may have absorbed some of the delayed demand for non-COVID-19 healthcare services. The shifts in healthcare utilization observed during this period highlight the critical role of public hospitals in responding to health crises, while also emphasizing the necessity of balancing healthcare resources between the public and private sectors to ensure service continuity during crises [55].

## 5. Conclusions

Our study reveals that the private hospital sector has experienced significant growth, with bed capacity, outpatient, and inpatient services share tripling compared to two decades ago. While the share of private sector in surgical procedures has increased by 2.5 times, the number of hospitalization days has sharply increased, quadrupling. However, this expansion has occurred alongside a more limited increase in the number of physicians (17%) and nurses/midwives (35%).

The growth of the private sector has been particularly pronounced in areas such as bed capacity, outpatient and inpatient admissions, surgical procedures, and hospitalization days. These expansion patterns appear to have been influenced by policy shifts, economic incentives, and extraordinary events such as the COVID-19 pandemic, especially when considering the identified structural breakpoints. Trend and breakpoint analyses indicate that policies encouraging private hospital expansion—implemented during the late 1990s—led to strong growth, while subsequent years starting from 2008 saw a slowdown due to regulatory measures aimed at restricting expansion. In some indicators, stabilization in the public-private sector shares is observed around 2010, while in general, stabilization becomes evident after 2012. Ultimately, we have clearly identified that policy changes have played a decisive role in shaping these transformations.

Policymakers must manage the expansion of the private healthcare sector in a way that ensures the capacity and sustainability of public healthcare services are maintained. In this regard, a strategic division of responsibilities, where the public sector focuses on more complex and high-cost healthcare services, while the private sector assumes a larger role in routine and elective care, could enhance resource efficiency. Furthermore, comprehensive and proactive strategic planning is essential to strengthen the resilience of the healthcare system against unexpected crises and ensure universal and equitable access to essential health services. Such an approach would contribute to maintaining a sustainable balance between public and private healthcare services.

In this context, reducing the patient-to-physician and patient-to-nurse ratios would enhance public healthcare capacity. This approach could help prevent deepening inequalities and mitigate the risks associated with the uncontrolled expansion of private healthcare services. Additionally, ensuring a resilient healthcare system and universal access to basic health services requires comprehensive and forward-looking strategic planning. We believe that such an approach will be instrumental in maintaining a sustainable balance between public and private healthcare services.

From a policy perspective, while the private sector has expanded its role in healthcare delivery, this growth must be balanced with strategies that ensure equitable access, particularly for disadvantaged populations. One way to achieve this is through regulatory mechanisms that prevent market-driven exclusion, such as setting price caps for essential services in private hospitals or offering financial incentives for private providers to expand services in underserved regions. Additionally, public–private partnership (PPP) models should be restructured to prioritize accessibility and affordability. While private sector participation can contribute to service expansion, it must be carefully managed to avoid exacerbating inequalities. Implementing targeted subsidy programs for low-income individuals, expanding telemedicine services to reduce regional disparities, and strengthening workforce retention policies for public hospitals could help mitigate the unintended consequences of privatization.

This study’s reliance on national and sectoral data presents certain limitations, particularly regarding its ability to capture regional disparities, urban-rural divides, and institutional differences in privatization outcomes. Future research should integrate province-level data on healthcare expenditures and facility distribution to provide a more detailed assessment of local healthcare accessibility. Furthermore, as our analysis is based on proportional trends, it does not directly assess the absolute increase in healthcare capacity. This study examines the 21-year trend in public and private sector shares within healthcare services. Additionally, socioeconomic factors such as income inequality, demographic changes, and macroeconomic conditions—which significantly influence healthcare demand and supply—were not explicitly accounted for in this analysis. Income disparities, along with variations in income levels, aging populations, and economic fluctuations, may have played a crucial role in shaping both private sector expansion and public healthcare utilization. Lastly, this study does not establish a direct causal relationship between privatization policies and observed changes. While the timing of structural breakpoints and policy changes were compared, external factors and random variations could have also contributed to these shifts.

To better evaluate the direct impact of privatization on healthcare accessibility and equity, future research should employ causal inference methods, such as difference-in-differences or instrumental variable approaches, to strengthen the robustness of findings and inform evidence-based policymaking.

## Figures and Tables

**Table 1:** Percentage distributions of various health service indicators between the public and private sectors (2002 to 2022).

Variables	Hospitals		Hospital Beds		Qualified Hospital Beds		Intensive Care Beds		Dialysis Machines		Outpatient Visits		Inpatients		Surgeries		Hospitalization Days		Physicians		Nurses and Midwives	
Sectors	Public (%)	Private (%)	Public (%)	Private (%)	Public (%)	Private (%)	Public (%)	Private (%)	Public (%)	Private (%)	Public (%)	Private (%)	Public (%)	Private (%)	Public (%)	Private (%)	Public (%)	Private (%)	Public (%)	Private (%)	Public (%)	Private (%)
2002	66.96	23.44	65.30	7.53	36.12	30.07	39.25	44.81	30.87	51.30	88.32	4.58	75.70	10.10	67.09	13.69	73.79	5.37	62.40	15.70	82.40	9.70
2003	57.00	20.99	65.13	7.81	36.13	31.42	41.63	40.04	32.62	51.71	87.82	4.75	74.59	10.79	66.73	13.99	73.03	5.55	61.30	16.80	82.10	10.00
2004	56.12	20.79	65.09	7.60	36.34	35.70	42.36	37.04	32.69	53.00	88.43	4.58	75.46	10.23	69.41	12.52	74.04	5.02	61.00	17.60	82.10	10.20
2005	66.30	24.50	64.40	8.12	41.24	33.80	42.66	35.71	33.48	54.24	88.00	5.86	72.27	13.03	66.66	15.49	70.74	6.61	59.80	19.10	81.80	10.30
2006	63.76	27.51	63.56	8.40	40.45	37.00	41.08	36.60	31.76	57.84	87.07	7.14	68.97	15.87	64.60	18.86	69.57	7.33	58.70	19.80	82.00	10.30
2007	64.39	27.71	62.94	9.77	41.94	36.55	43.89	33.22	30.96	60.11	84.14	9.83	65.08	20.44	62.20	22.08	69.10	8.35	57.40	21.30	80.00	11.70
2008	62.74	29.63	62.47	11.43	48.18	32.75	45.98	31.62	29.32	62.79	79.18	14.14	61.54	24.14	58.82	25.95	66.45	10.58	56.20	22.70	77.00	13.10
2009	60.04	32.40	61.20	13.35	47.21	33.94	45.89	32.35	28.91	63.61	77.31	16.13	59.94	25.29	52.16	32.25	66.41	12.08	56.50	22.50	75.50	14.40
2010	58.58	33.98	60.02	14.01	52.20	31.01	45.00	34.65	28.73	64.05	77.62	15.75	60.42	25.24	53.23	31.72	65.69	12.61	58.70	20.70	74.50	15.60
2011	57.81	34.62	62.36	16.27	50.29	30.93	45.67	35.78	28.98	64.04	75.28	17.48	59.24	26.70	52.60	32.70	65.34	13.88	58.20	20.80	77.00	12.70
2012	56.10	36.48	61.14	17.88	49.34	32.27	43.57	38.55	28.67	63.17	73.59	18.77	57.53	29.09	52.13	32.80	62.43	16.84	56.80	22.40	74.60	14.20
2013	56.30	36.26	60.02	18.80	48.89	32.83	42.19	38.88	29.70	63.21	73.25	18.83	56.76	30.06	51.55	33.17	63.02	16.94	56.40	22.40	73.80	14.80
2014	56.68	36.39	59.80	19.59	51.35	29.72	41.56	40.49	30.87	61.79	73.66	18.24	56.74	29.92	50.96	33.09	61.85	18.36	57.40	21.80	73.00	15.20
2015	56.43	36.66	58.35	20.82	52.49	29.66	39.84	43.10	31.24	61.31	73.30	18.45	54.71	31.31	49.57	33.63	60.13	20.00	58.50	21.10	72.80	15.90
2016	58.01	37.42	61.04	21.65	55.00	27.42	41.21	42.40	32.54	60.23	75.97	15.89	56.21	30.10	51.83	31.43	61.54	19.15	59.60	19.20	73.60	15.00
2017	57.91	37.62	59.92	21.78	56.98	25.24	41.38	42.44	33.64	59.03	76.09	15.53	55.48	30.06	52.53	30.94	60.65	19.64	60.20	19.70	73.30	15.40
2018	57.95	37.61	60.22	21.64	59.74	23.68	42.22	41.93	34.40	58.90	76.27	15.10	56.23	29.44	53.19	29.45	61.18	20.00	59.80	19.20	72.70	15.20
2019	58.19	37.39	60.38	21.54	61.47	22.41	43.52	40.96	35.08	58.38	76.53	14.35	56.08	28.91	53.53	28.30	61.27	19.72	60.40	18.60	72.60	14.80
2020	58.67	36.90	62.49	20.79	63.46	21.41	50.19	35.42	35.66	57.81	72.15	18.31	51.95	33.49	43.36	37.60	59.46	22.19	60.80	17.90	73.90	13.50
2021	58.69	36.91	61.97	21.14	63.38	21.41	49.77	35.88	36.77	56.81	73.60	16.72	51.85	32.38	46.58	33.44	58.69	22.03	58.40	20.00	72.20	14.70
2022	58.84	36.78	62.25	21.00	64.10	20.91	49.46	36.15	37.06	56.44	75.72	15.07	53.97	30.63	51.92	29.42	60.12	20.61	60.10	18.30	74.40	13.10

**Table 2:** Trend tests and difference statistics for variables in the public and private healthcare sectors.

Proportional Variables	Public			Private			Z-Diff.	*p*
	z	τ	*p*	z	τ	*p*		
All PV	−1.63	−0.258	0.1040	2.35	0.373	0.0187 *	4.516	1.33 × 10^−15^ *
H&HB	−2.51	−0.400	0122 *	5.10	0.809	3.338 × 10^−7^ *	5.380	7.41 × 10^−8^ *
PI	3.53	0.562	4.11 × 10^−4^ *	2.567	0.410	0.0102 *	0.683	0.4944
Hospitals	−0.634	−0.105	0.526	4.92	0.781	8.56 × 10^−7^ *	3.93	8.54 × 10^−5^ *
Hospital Beds	−3.05	−0.486	0.002 *	5.10	0.810	3.34 × 10^−7^ *	5.77	8.16 × 10^−9^ *
Qualified Hospital Beds	5.71	0.905	1.15 × 10^−8^ *	−4.38	−0.695	1.19 × 10^−5^ *	7.13	9.91 × 10^−13^ *
Intensive Care Beds	1.36	0.220	0.174	0.755	0.124	0.450	0.427	0.6690
Dialysis Machines	2.45	−0.391	0.014 *	−0.030	−0.010	0.976	1.75	0.0799
HU	−4.74	−0.752	8.56 × 10^−7^ *	4.26	0.676	2.06 × 10^−5^ *	6.46	1.98 × 10^−10^ *
Outpatient Visits	3.65	−0.581	1.10 × 10^−4^ *	2.66	0.425	0.008 *	4.64	8.06 *
Inpatients	−5.65	−0.895	1.63 × 10^−8^ *	4.92	0.781	8.56 × 10^−7^ *	7.47	7.82 × 10^−9^ *
Surgeries	−4.02	−0.639	5.91 × 10^−5^ *	3.17	0.505	0.002 *	5.08	3.73 × 10^−7^ *
Hospitalization Days	−5.59	−0.895	2.32 × 10^−8^ *	5.71	0.905	1.15 × 10^−8^ *	7.99	1.33 × 10^−9^ *
HW	−2.90	−0.465	0.0036 *	1.72	0.278	0.0849	3.27	1.07 × 10^−3^ *
Physicians	0.000	0.005	1.000	−0.091	−0.019	0.928	0.064	0.9488
Nurses/Midwives	−4.81	−0.766	1.54 × 10^−6^ *	3.12	0.500	0.002 *	5.60	2.12 × 10^−8^ *

PV, proportional variables (all); H&HB, hospitals and hospital beds; PI, physical investment; HU, healthcare utilization; HW, healthcare workforce; *: *p* < 0.05.

**Table 3:** Analysis of trend changes based on structural breaks using segmented regression models.

		Models	β	SE	t	*p*
General (All Variables)	Public	Intercept ^a^	5.7595373	1.2542574	4.592	0.000199 *
		Years ^a^	−0.0025720	0.0006234	−4.126	0.000575 *
		U1-Breakpoint (2008)	0.0103504	0.0010273	1.07	1.39 × 10^−8^ *
		^a^: Newey–West estimator; df: 17; F: 17.02; *p* = 0.0005748; R^2^: 0.4726 and adjusted R^2^: 0.4448				
	Private	Intercept ^a^	−9.4028199	1.6206106	−5.802	1.37 × 10^−5^ *
		Years ^a^	0.004803	0.0008055	5.964	9.70 × 10^−6^ *
		U1-Breakpoint (2005)	0.0164313	0.0035258	4.660	0.0002 *
		U2-Breakpoint (2008)	−0.0083473	0.0023082	−3.616	0.0021 *
		U3-Breakpoint (2011)	−0.0100074	0.0014394	−6.952	2.33 × 10^−6^ *
		^a^: Newey–West estimator; df: 13; F: 35.56; *p* = 9.702 × 10^−6^; R^2^: 0.6518 and adjusted R^2^: 0.6335				
Hospitals and Hospital Beds	Public	Intercept ^a^	5.3710283	10.2808635	0.5224	0.6074
		Years ^a^	−0.0023680	0.0051304	−0.4616	0.6496
		U1-Breakpoint (2008)	0.009080	0.002470	3.676	0.00187 *
		^a^: Newey–West estimator; df: 17; F: 11.94; *p* = 0.002647343; R^2^: 0.3859715 and adjusted R^2^: 0.3499				
	Private	Intercept ^a^	−17.0964123	6.2524081	−2.7344	0.01317 *
		Years ^a^	0.0086171	0.0031057	2.7746	0.01207 *
		U1-Breakpoint (2008)	0.024939	0.008181	3.048	0.0111 *
		U2-Breakpoint (2011)	0.005851	0.005836	1.002	0.3379
		U3-Breakpoint (2015)	−0.010535	0.001507	−6.991	2.30 × 10^−5^ *
		U4-Breakpoint (2019)	−0.007374	0.001829	−4.031	0.00198 *
		^a^: Newey–West estimator; df: 11; F: 126.71; *p* = 7.572117 × 10^−10^; R^2^: 0.8696018 and adjusted R^2^: 0.8577				
Physical Investment	Public	Intercept ^a^	5.759537	6.154448	0.9358	0.3611
		Years ^a^	−0.002572	0.003060	−0.841	0.4111
		U1-Breakpoint (2004)	0.015931	0.011228	1.419	0.1836
		U2-Breakpoint (2011)	−0.009180	0.003675	−2.498	0.0296 *
		U3-Breakpoint (2015)	0.014515	0.002307	6.292	5.90 × 10^−5^ *
		U4-Breakpoint (2019)	−0.009019	0.010858	−0.831	0.4236
		^a^: Newey–West estimator; df: 11 F: 28.28; *p* = 3.926051 × 10^−5^; R^2^: 0.472558 and adjusted R^2^: 0.4447979				
	Private	Intercept ^a^	−9.4028199	8.6919303	−1.082	0.2929
		Years ^a^	0.0048035	0.0043144	1.1134	0.2794
		U1-Breakpoint (2005)	0.020309	0.009746	2.084	0.0613
		U2-Breakpoint (2008)	−0.003415	0.006953	−0.491	0.6331
		U3-Breakpoint (2011)	−0.004980	0.003567	−1.396	0.1903
		U4-Breakpoint (2016)	−0.010437	0.003775	−2.765	0.0184 *
		U5-Breakpoint (2019)	0.005925	0.007062	0.839	0.4193
		^a^: Newey–West estimator; df: 9; F: 12.94; *p* = 0.001920514; R^2^: 0.6517855 and adjusted R^2^: 0.6334584				
Healthcare Utilization	Public	Intercept ^b^	8.2529810	8.8192971	0.936	0.3618
		Years ^b^	−0.0037907	0.0043710	−0.867	0.3972
		U1-Breakpoint (2004)	−0.025300	0.008822	−2.868	0.0107 *
		U2-Breakpoint (2007)	−0.030030	0.009672	−2.834	0.0064 *
		U3-Breakpoint (2011)	0.023520	0.004443	5.294	0.00006 *
		U4-Breakpoint (2019)	0.025960	0.006323	4.106	0.00074 *
		^b^: Cochran–Orcutt estimator; df: 19 F: 0.80; *p* = 0.3972; R^2^: 0.0401 and adjusted R^2^: 0.0132				
	Private	Intercept ^a^	−18.4437580	12.6577409	−1.457	0.1614
		Years ^a^	0.0092662	0.0062892	1.4733	0.1570
		U1-Breakpoint (2004)	0.029997	0.010714	2.800	0.0173 *
		U2-Breakpoint (2007)	−0.01806	0.008099	−2.230	0.0475 *
		U3-Breakpoint (2011)	−0.009753	0.005898	−1.654	0.1264
		^a^: Newey–West estimator; df: 13; F: 62.73; *p* = 1.945415 × 10^−7^; R^2^: 0.7675136 and adjusted R^2^: 0.7552775				
Healthcare Workforce	Public	Intercept ^b^	0.23765250	3.76066574	0.063	0.9503
		Years ^b^	0.00021141	0.00186510	0.113	0.9110
		U1-Breakpoint (2004)	−0.016000	0.010842	−1.476	0.156
		U2-Breakpoint (2007)	0.021366	0.010592	2.017	0.058
		^b^: Cochran–Orcutt estimator; df: 15 F: 0.10; *p* = 0.911; R^2^: 0.0007 and adjusted R^2^: −0.0548				
	Private	Intercept ^b^	4.5364067	3.5686851	1.271	0.2199
		Years ^b^	−0.0021629	0.001769	−1.222	0.2374
		U1-Breakpoint (2004)	0.007557	0.005160	1.465	0.171
		U2-Breakpoint (2007)	−0.012800	0.004777	−2.679	0.021 *
		U3-Breakpoint (2015)	−0.003710	0.002107	−1.761	0.106
		^b^: Cochran–Orcutt estimator; df: 13; F: 1.50; *p* = 0.02374; R^2^: 0.0766 and adjusted R^2^: 0.0253				

*, *p* < 0.05.

## Data Availability

The original contributions presented in this study are included in the article/Supplementary Materials. Further inquiries can be directed to the corresponding author.

## Supplementary Materials

The following supporting information can be downloaded at https://www.mdpi.com/article/10.3390/healthcare13060622/s1, Figure S1: Physical Investment. Figure S2: Healthcare Utilization. Figure S3: Healthcare workforce. Table S1: Dataset.
