# Supplementary material for: Healthcare Sector Dynamics in Turkey (2002–2022): Trends, Breakpoints, and Policy Implications (Privatization in the Hospital Sector)
Source: Healthcare (Basel). 2025 Mar 13;13(6):622. doi: 10.3390/healthcare13060622 (PMC11942332; doi:10.3390/healthcare13060622)
Supplement: Supplementary file 1 [file healthcare-13-00622-s001.zip › healthcare-3495108-supplementary.pdf]

Supplementary file
Findings and Healthcare Services Indicators

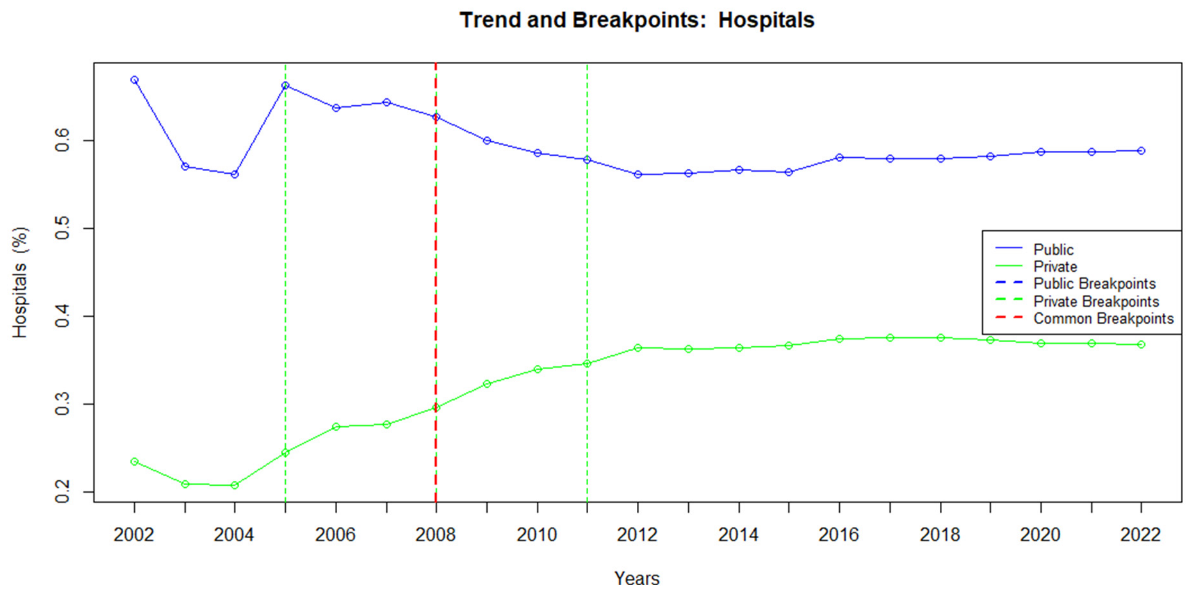

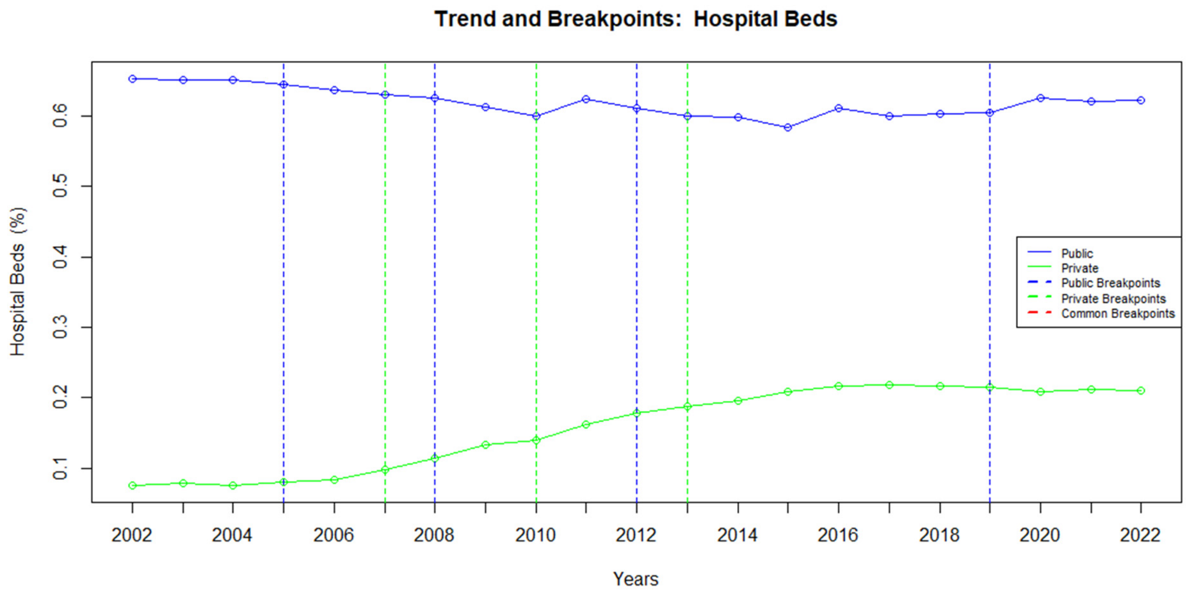

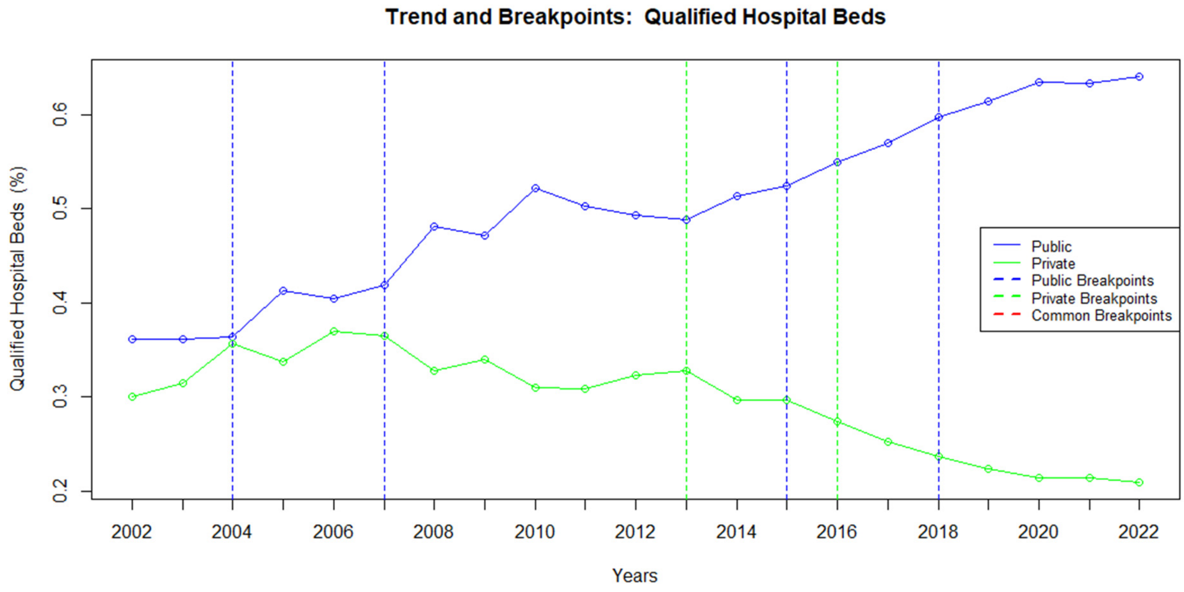
Trend and Breakpoints: Qualified Hospital Beds
Qualified Hospital Beds (%)
0.2
0.3
0.4
0.5
0.6
2002
2004
2006
2008
2010
2012
2014
2016
2018
2020
2022
Years
Public
Private
Public Breakpoints
Private Breakpoints
Common Breakpoints

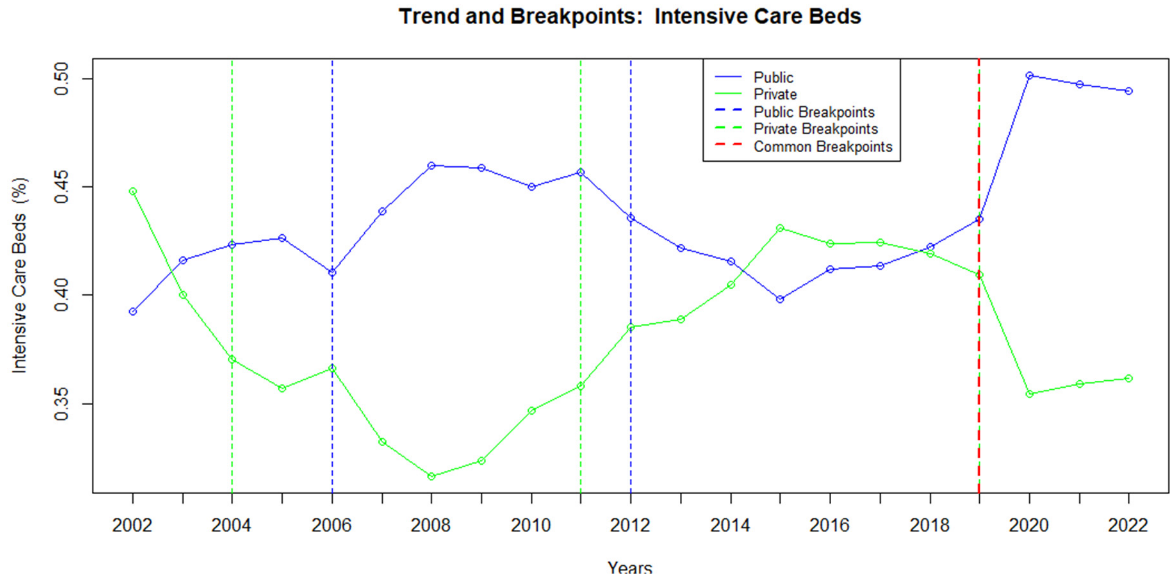
Trend and Breakpoints: Intensive Care Beds
Intensive Care Beds (%)
0.35
0.40
0.45
0.50
2002
2004
2006
2008
2010
2012
2014
2016
2018
2020
2022
Years
Public
Private
Public Breakpoints
Private Breakpoints
Common Breakpoints

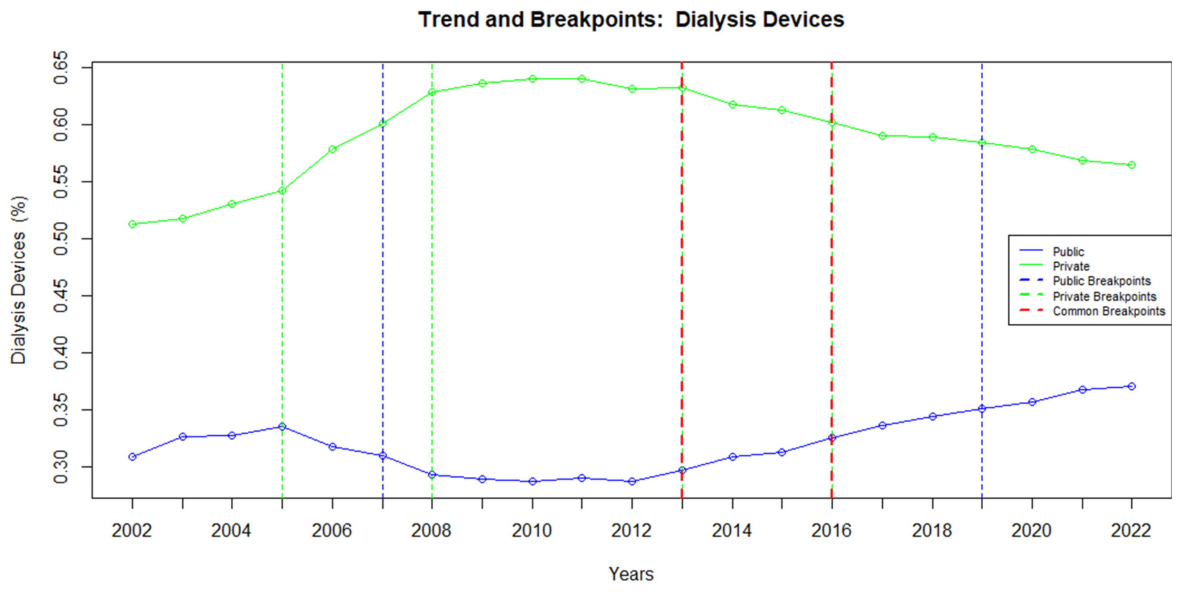

Figure S1. Physical Investment

For hospitals, structural breakpoints were observed in **2005, 2008, and 2011** in the **private sector**, whereas in the **public sector**, a **single breakpoint occurred in 2008**. In the **private sector**, the **2005 and 2008 breakpoints** marked an **accelerated increase**, reflecting the rapid expansion of private hospitals, while the **2011 breakpoint** indicated a **moderate growth rate**. In contrast, the **2008 breakpoint in the public sector** represented a **decline in public hospital expansion**, signaling a shift in healthcare investments toward privatization. For hospital beds, the structural breakpoints occurred in 2005, 2008, 2012, and 2019 in the public sector, whereas in the private sector, the breakpoints were observed in 2007, 2010, and 2013. In the public sector, the breakpoints in 2005 and 2008 exhibited an accelerated decline, whereas those in 2012 and 2019 showed a slow decline rate. Conversely, in the private sector, the 2007 and 2010 breakpoints showed an accelerated increase, whereas the 2013 breakpoint reflected a slower growth rate. Regarding qualified hospital beds, the public sector experienced considerable changes in 2004, 2007, 2015, and 2018, whereas the private sector exhibited structural breakpoints in 2013 and 2016. In the public sector, the breakpoints in 2004, 2007, and 2015 exhibited an accelerated growth rate, but the 2018 breakpoint showed a slow decline rate. In the private sector, the 2013 breakpoint reflected an increased rate of decline, whereas the 2016 breakpoint indicated a slower decline. For intensive care beds, the structural breakpoints in the public sector occurred in 2006, 2012, and 2019, whereas the private sector experienced changes in 2004, 2011, and 2019. In the public sector, the 2006 breakpoint showed an accelerated growth, the 2012 breakpoint reflected a shift to a declining trend, and the 2019 breakpoint indicated renewed growth. In the private sector, the 2004 breakpoint showed a slow decline rate, the 2011 breakpoint exhibited a shift to growth, and the 2019 breakpoint reflected a return to decline. Finally, for dialysis devices, significant breakpoints occurred in 2007, 2013, 2016, and 2019 in the public sector and in 2005, 2008, 2013, and 2016 in the private sector. In the public sector, the 2007 breakpoint reflected a slowdown in growth, the 2013 breakpoint exhibited a shift to a declining trend, the 2016 breakpoint indicated a slower decline rate, and the 2019 breakpoint showed an accelerated decline. In the private sector, the 2005 breakpoint exhibited a shift to a declining trend, the 2008 breakpoint showed a slowdown and stabilization, the 2013 breakpoint reflected a shift to growth, and the 2016 breakpoint indicated an accelerated growth rate (**Figure** S1).

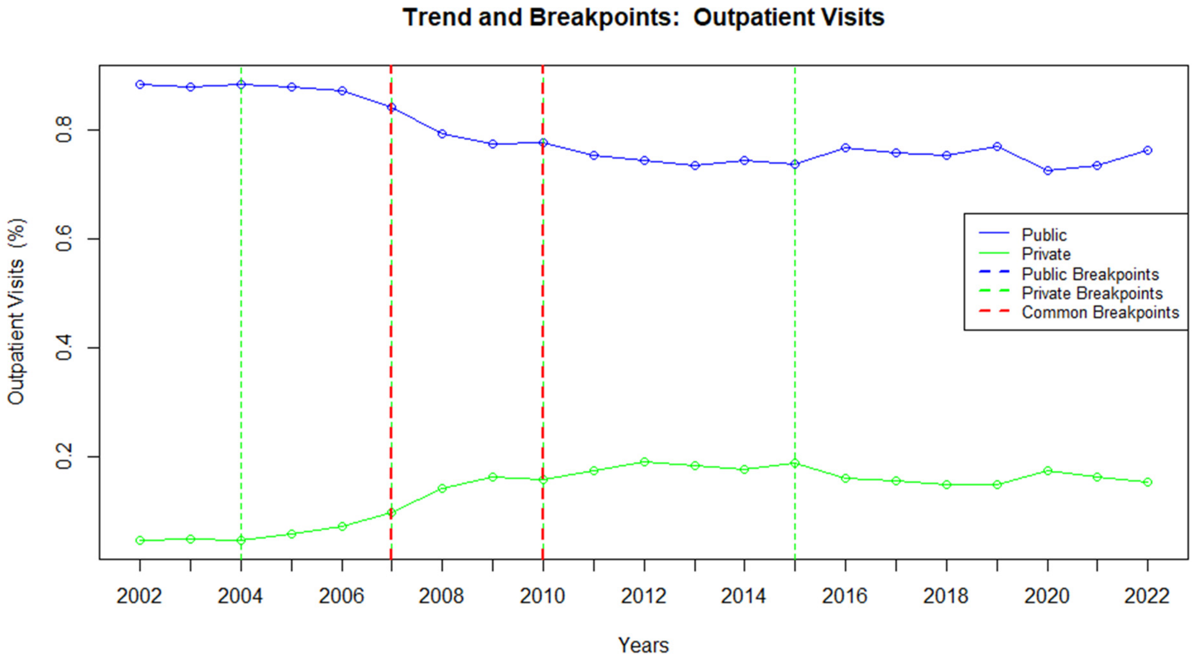
Trend and Breakpoints: Outpatient Visits
Outpatient Visits (%)
0.2
0.4
0.6
0.8
2002
2004
2006
2008
2010
2012
2014
2016
2018
2020
2022
Years
Public
Private
Public Breakpoints
Private Breakpoints
Common Breakpoints

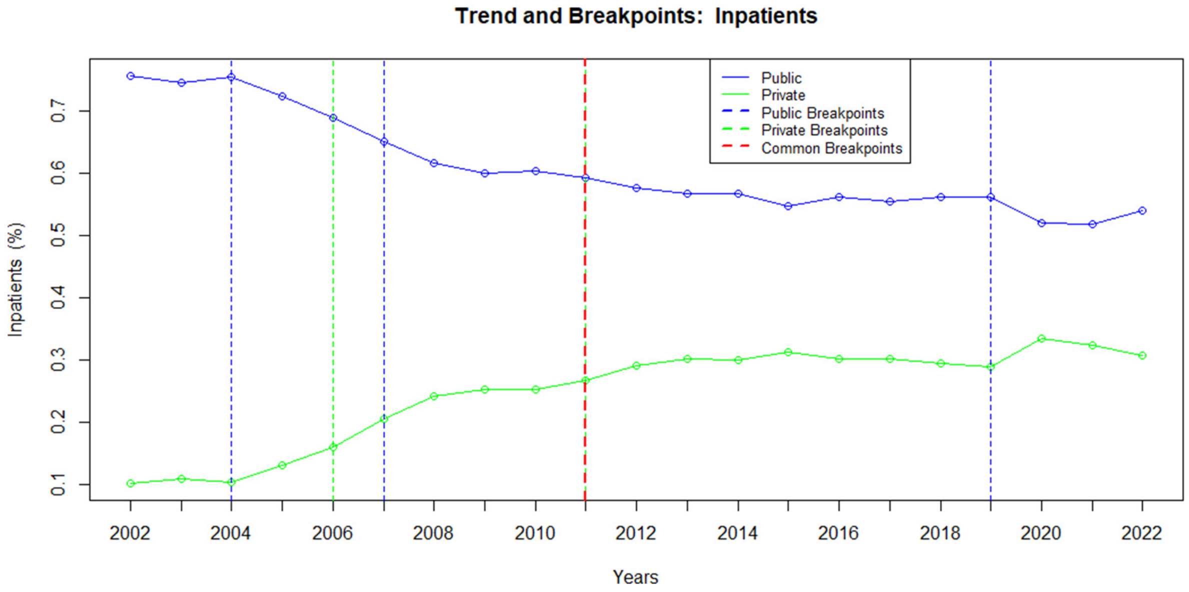
Trend and Breakpoints: Inpatients
Inpatients (%)
0.1
0.2
0.3
0.4
0.5
0.6
0.7
2002
2004
2006
2008
2010
2012
2014
2016
2018
2020
2022
Years
Public
Private
Public Breakpoints
Private Breakpoints
Common Breakpoints

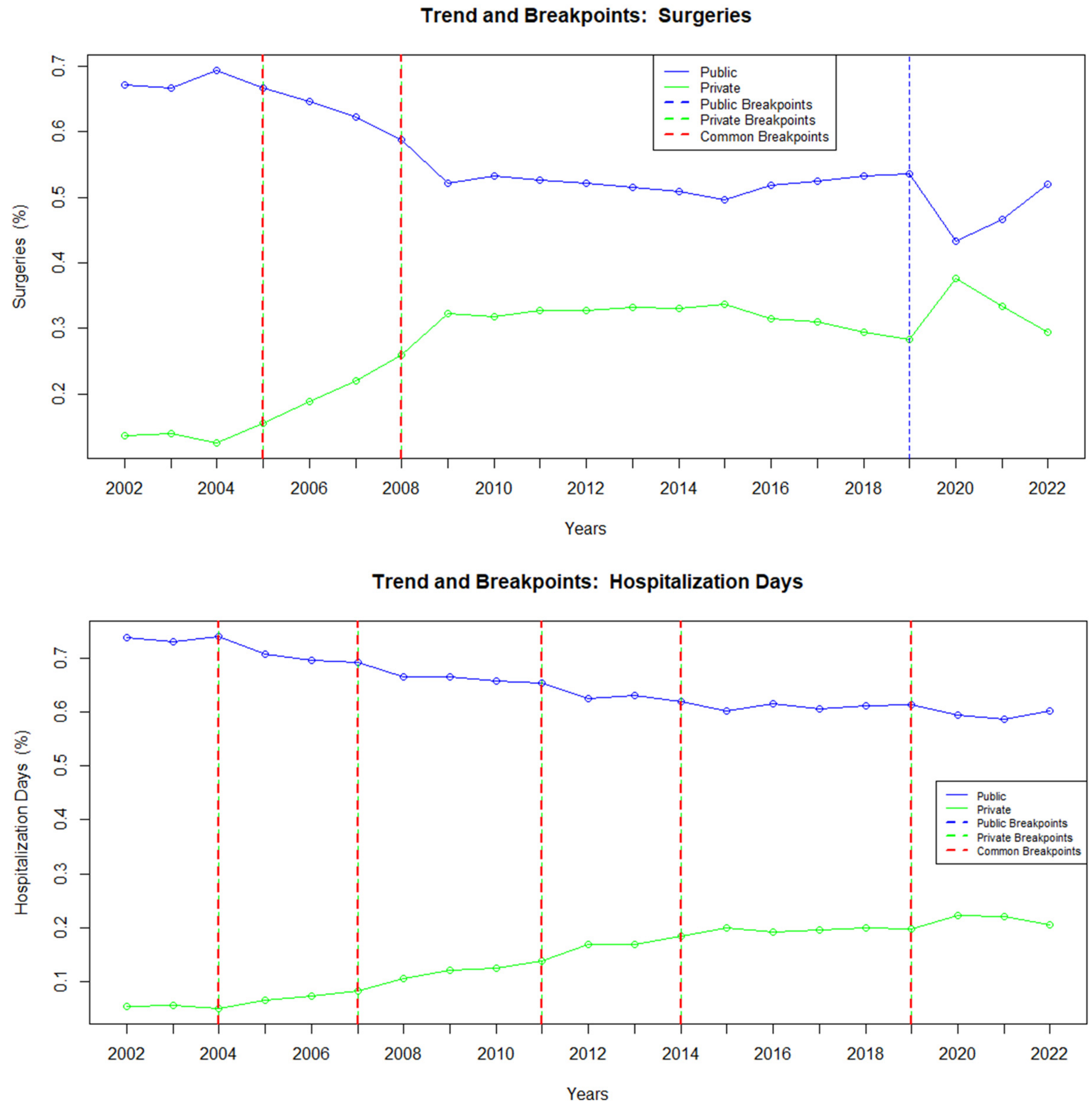

Figure S2. Healthcare Utilization

For outpatient visits, the public sector showed a declining trend after the 2007 breakpoint, which stabilized in 2010. In the private sector, a shift to an increasing trend was observed in 2004, with a deceleration in 2007, stabilization with a slight growth in 2010, and a minor declining trend after the 2015 breakpoint. For inpatient cases, the public sector demonstrated a rapidly declining trend starting in 2004, which slowed down in 2007, stabilized in 2011, and declined after 2019. In contrast, the private sector experienced a slowdown in its increasing trend for inpatient cases after the 2011 breakpoint.
For surgeries, the public sector showed a declining trend in 2005, stabilization in 2008, and an intensified decline in 2019. In the private sector, the trend for surgeries increased in 2005 but stabilized in 2008.
Regarding hospital days, the public sector displayed a declining trend after the 2004 breakpoint, which further declined in 2007, decelerated in 2011, nearly stabilized in 2014, and slightly declined in 2019. In the private sector, hospital days showed an increasing trend after the 2004 breakpoint, which accelerated in 2007, decelerated in 2011, and slightly increased in 2019 (**Figure** S2).

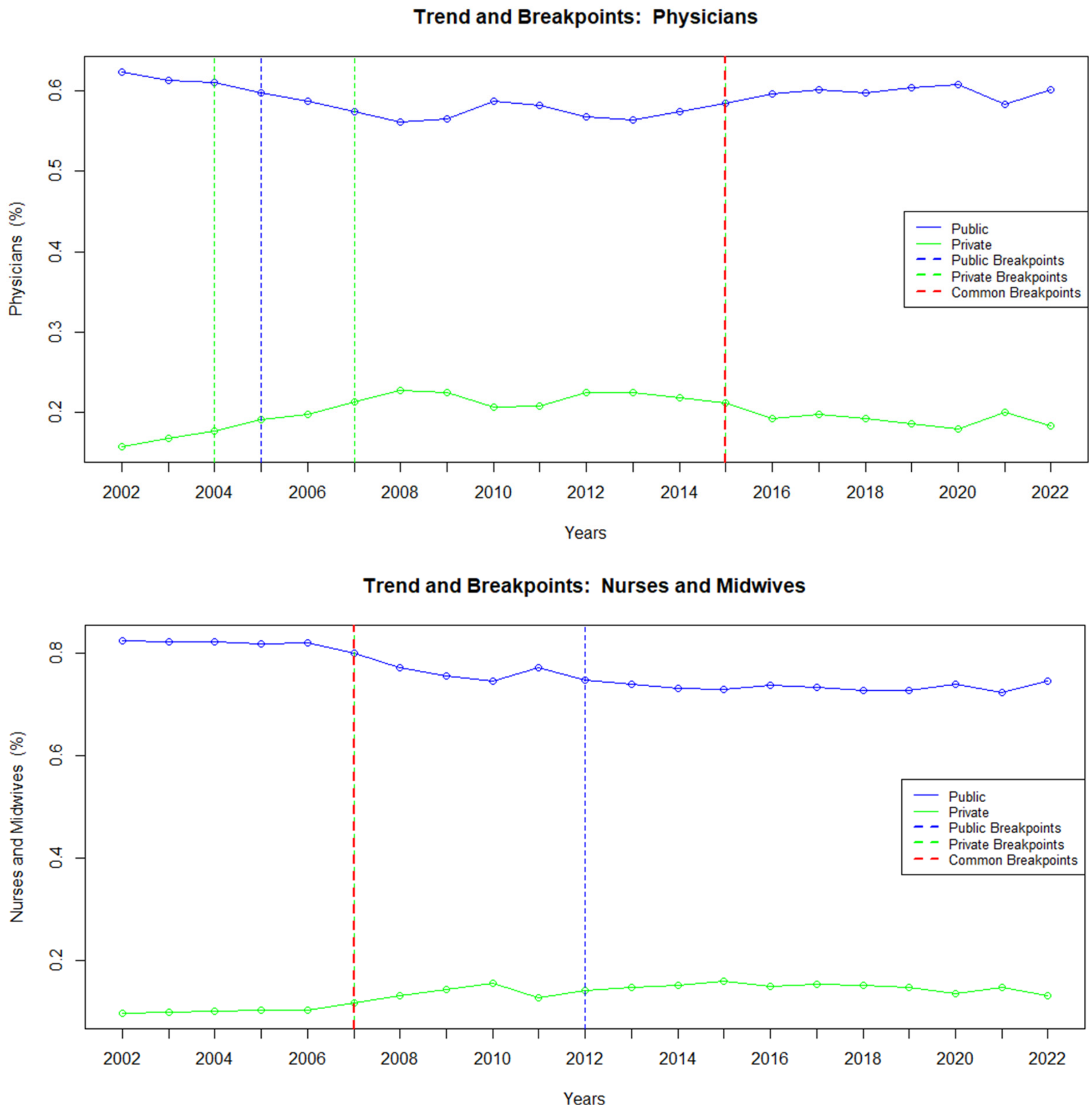

Figure S3. Healthcare workforce

For the ratio of doctors, the public sector experienced two breakpoints: one in 2005, showing stabilization, and another in 2015, marking the start of an increasing trend. In the private sector, the ratio of doctors demonstrated an increasing trend at the 2004 breakpoint, stabilization at the 2007 breakpoint, and a declining trend after the 2015 breakpoint. For the ratio of nurses/midwives, the public sector had breakpoints in 2007 and 2012. The 2007 breakpoint showed a declining trend, whereas the 2012 breakpoint displayed stabilization. In the private sector, only one breakpoint was identified in 2007, reflecting stabilization (**Figure** S3).

The findings of the Mann-Kendall relationship analysis based on variables are as follows:

- **Ratio of Hospitals**: The public sector showed a weak, negative, and insignificant trend ($\tau = -0.105$, $P = 0.526$), whereas the private sector displayed a strong, positive, and significant trend ($\tau = 0.781$, $P = 8.56e-07$). The difference between the sectors was significant ($P = 8.54e-05$).

- **Ratio of Hospital Beds**: The public sector showed a strong, negative, and significant trend ($\tau = -0.486$, $P = 2.29e-03$), whereas the private sector showed a strong, positive, and significant trend ($\tau = 0.810$, $P = 3.34e-07$). The difference between the sectors was significant ($P = 8.16e{-}09$).
- **Ratio of Qualified Beds**: The public sector displayed a strong, positive, and significant trend ($\tau = 0.905$, $P = 1.15e-08$), whereas the private sector exhibited a strong, negative, and significant trend ($\tau = -0.695$, $P = 1.19e-05$). The difference between the sectors was significant ($P = 9.91e-13$).
- **Ratio of Intensive Care Beds**: None of the sectors showed a significant trend (public: $\tau = 0.220$, $P = 0.174$; private: $\tau = 0.124$, $P = 0.450$), and the difference between them was insignificant ($P > 0.05$).
- **Ratio of Dialysis Devices**: The public sector exhibited a moderate, positive, and significant trend ($\tau = 0.391$, $P = 0.0144$), whereas the private sector showed no significant trend ($P = 0.976$). The difference between the sectors was not significant ($P > 0.05$).
- **Outpatient Visits**: The public sector showed a strong, negative, and significant trend ($\tau = -0.581$, $P = 1.10e-04$), whereas the private sector demonstrated a moderate, positive, and significant trend ($\tau = 0.425$, $P = 0.00785$). The difference between the sectors was significant ($P = 3.95e-06$).
- **Inpatient Cases**: The public sector displayed an excellent, negative, and significant trend ($\tau = -0.895$, $P = 1.63e-08$), whereas the private sector showed a strong, positive, and significant trend ($\tau = 0.781$, $P = 8.56e-07$). The difference between the sectors was significant ($P = 7.82e-14$).
- **Surgeries**: The public sector exhibited a strong, negative, and significant trend ($\tau = -0.639$, $P = 5.91e-05$), whereas the private sector displayed a strong, positive, and significant trend ($\tau = 0.505$, $P = 0.0015$). The difference between the sectors was significant ($P = 3.74e-07$).
- **Hospital Days**: The public sector showed an excellent, negative, and significant trend ($\tau = -0.895$, $P = 2.32e-08$), whereas the private sector displayed an excellent, positive, and significant trend ($\tau = 0.905$, $P = 1.15e{-}08$). The difference between the sectors was significant ($P = 1.33e-15$).
- **Ratio of Doctors**: None of the sectors showed a significant trend (public: $\tau = 0.005$, $P = 1.000$; private: $\tau = -0.019$, $P = 0.928$), and the difference between the sectors was not significant ($P > 0.05$).
- **Ratio of Nurses/Midwives**: The public sector demonstrated a strong, negative, and significant trend ($\tau = -0.766$, $P = 1.54e-06$), whereas the private sector exhibited a strong, positive, and significant trend ($\tau = 0.500$, $P = 0.002$). The difference between the sectors was significant ($P = 2.13e-08$) (Table 2).

Broken-stick regression models were used to interpret the changes in the slopes following the breakpoints in the trends. The assumptions for the segmented regression were examined as follows:

- **Normality:** The Shapiro–Wilk test was conducted, and normality was confirmed for all cases ($W_{public} = 0.95167$, $p_{public} = 0.3662$; $W_{private} = 0.95941$, $p_{private} = 0.5043$; $W_{H\&HBPublic} = 0.93446$, $p_{H\&HBpublic} = 0.1691$; $W_{H\&HBprivate} = 0.9573$, $p_{H\&HBprivate} = 0.4635$; $W_{PIpublic} = 0.9611$, $p_{PIpublic} = 0.5387$; $W_{PIprivate} = 0.94507$, $p_{PIprivate} = 0.274$; $W_{HUpublic} = 0.92904$, $p_{HUpublic} = 0.1317$; $W_{HUprivate} = 0.91916$, $p_{HUprivate} = 0.08348$; $W_{HWpublic} = 0.93261$, $p_{HWpublic} = 0.1553$; $W_{HWprivate} = 0.92908$, $p_{HWprivate} = 0.1319$).

- **Homoskedasticity:** The Breusch–Pagan test was conducted to assess the absence of heteroskedasticity. Homoskedasticity was confirmed in most cases ($BP_{public} = 0.71432$, $p_{public} = 0.398$; $BP_{private} = 0.29764$, $p_{private} = 0.5854$; $BP_{H\&HBpublic} = 2.6264$, $p_{H\&HBpublic} = 0.1051$ *[as heteroskedasticity was detected in H&HB for public in the first stage, generalized least squares was applied instead of ordinary least squares]*; $BP_{H\&HBprivate} = 11.454$, $p_{H\&HBprivate} = 0.2459$; $BP_{PIpublic} = 2.5019$, $p_{PIpublic} = 0.1137$; $BP_{PIprivate} = 3.6295$, $p_{PIprivate} = 0.05676$; $BP_{HUpublic} = 0.002989$, $p_{HUpublic} = 0.9564$; $BP_{HUprivate} = 0.0029399$, $p_{HUprivate} = 0.9568$; $BP_{HWpublic} = 0.40206$, $p_{HWpublic} = 0.526$; $BP_{HWprivate} = 0.69712$, $p_{HWprivate} = 0.4038$).

- **Autocorrelation**: The presence of autocorrelation was tested using the Durbin–Watson test. Autocorrelation was detected and corrected using the Cochran–Orcutt and Newey–West estimators

for the public and private sectors. Residuals were re-evaluated, confirming the resolution of autocorrelation issues for the corrected variables (DW_{public}: 2.11235, p_{public} = 0.4998; $DW_{private}$: 1.655136, $p_{private}$ = 0.145615; $DW_{HUpublic}$: 1.72159; $p_{HUpublic}$ = 0.1841; $DW_{HWpublic}$: 2.01860, $p_{HWpublic}$ = 0.4148; $DW_{HWprivate}$: 2.37786, $p_{HWprivate}$ = 0.145615).

Table S1. Dataset

Yillar	Kamu_Hastane_Sayisi	Ozel_Hastane_Sayisi	Kamu_Hastane_Yatagi_Sayisi	Ozel_Hastane_Yatagi_Sayisi	Kamu_Nitelikli_Hastane_Yatagi_Sayisi	Ozel_Nitelikli_Hastane_Yatagi_Sayisi	Kamu_Yogun_Bakim_Yatagi_Sayisi	Ozel_Yogun_Bakim_Yatagi_Sayisi	Kamu_Diyaliz_Cihazi_Sayisi	Ozel_Diyaliz_Cihazi_Sayisi	Kamu_Poliklinik_Basvurusu	Ozel_Poliklinik_Basvurusu	Kamu_Yatan_Hasta_Sayisi	Ozel_Yatan_Hasta_Sayisi	Kamu_Ameliyat_Sayilari	Ozel_Ameliyat_Sayilari	Kamu_Hastanede_Yatilan_Gun_Sayisi	Ozel_Hastanede_Yatilan_Gun_Sayisi	Kamu_Hekim_Sayisi	Ozel_Hekim_Sayisi	Kamu_Hemsire_ve_Ebe_Sayisi	Ozel_Hemsire_ve_Ebe_Sayisi
2002	0,6696	0,2344	0,652966176	0,075314189	0,36120207	0,300676033	0,392502258	0,448057814	0,308730321	0,51298303	0,8832	0,0458	0,757004341	0,101028945	0,670947508	0,13691329	0,737871465	0,05372135	0,624	0,157	0,824	0,097
2003	0,5700	0,2099	0,651322032	0,078064848	0,361325188	0,314191729	0,41634981	0,400427757	0,326217938	0,517128594	0,8782	0,0475	0,745890386	0,107906801	0,667321967	0,139902089	0,730324487	0,055472857	0,613	0,168	0,821	0,1
2004	0,5612	0,2079	0,65090848	0,076007606	0,363407062	0,357043073	0,423597078	0,370433701	0,326928471	0,530014025	0,8843	0,0458	0,754631878	0,102259347	0,694085614	0,125195472	0,740374318	0,050157354	0,61	0,176	0,821	0,102
2005	0,6630	0,2450	0,644017734	0,081159488	0,412432072	0,337973115	0,426619606	0,357053399	0,334762926	0,542411246	0,88	0,0586	0,722721343	0,130278068	0,666640761	0,154912391	0,707443836	0,066072771	0,598	0,191	0,818	0,103
2006	0,6376	0,2751	0,63564144	0,083967145	0,404549868	0,370037453	0,410750137	0,366033948	0,317576961	0,578351539	0,8707	0,0714	0,689749636	0,158695245	0,646016259	0,188647401	0,695680755	0,073275879	0,587	0,198	0,82	0,103
2007	0,6439	0,2771	0,629421348	0,097735955	0,419447808	0,365512231	0,438881162	0,332174316	0,309627918	0,601105519	0,8414	0,0983	0,65077373	0,204394717	0,621997849	0,220841602	0,691001642	0,08350021	0,574	0,213	0,8	0,117
2008	0,6274	0,2963	0,624664953	0,114301	0,481753985	0,327519134	0,459792338	0,316219119	0,293176742	0,627927084	0,7918	0,1414	0,615389739	0,241444842	0,588235392	0,25946496	0,664498656	0,105760756	0,562	0,227	0,77	0,131
2009	0,6004	0,3240	0,611981679	0,133472577	0,472066934	0,339417702	0,45886392	0,323533084	0,289112597	0,636144246	0,7731	0,1613	0,599392223	0,252886082	0,521604058	0,322505532	0,66413945	0,120833711	0,565	0,225	0,755	0,144
2010	0,5858	0,3398	0,600182782	0,140147524	0,521983558	0,310076953	0,449997269	0,346496259	0,287289454	0,640533045	0,7762	0,1575	0,604199418	0,252424898	0,532280426	0,317213678	0,656857456	0,126053692	0,587	0,207	0,745	0,156
2011	0,5781	0,3462	0,623622136	0,162711307	0,502877566	0,309331722	0,456738332	0,35782047	0,289826014	0,640385486	0,7528	0,1748	0,592400432	0,267047607	0,525957743	0,327044118	0,653428693	0,138800308	0,582	0,208	0,77	0,127
2012	0,5610	0,3648	0,6113899	0,178770643	0,49336725	0,322710631	0,43568745	0,385495378	0,286710156	0,631683295	0,7359	0,1877	0,57533655	0,290937669	0,521265162	0,328017799	0,624339596	0,168413078	0,568	0,224	0,746	0,142
2013	0,5630	0,3626	0,600249467	0,188005801	0,488869919	0,328283374	0,421853335	0,388769065	0,297014832	0,632079604	0,7325	0,1883	0,567606631	0,300623337	0,515460255	0,331710372	0,630230347	0,169417557	0,564	0,224	0,738	0,148
2014	0,5668	0,3639	0,598010018	0,195850819	0,513464135	0,297225972	0,415581688	0,404906902	0,308702689	0,617903386	0,7366	0,1824	0,567445457	0,29924239	0,509575228	0,330900369	0,618549334	0,183602588	0,574	0,218	0,73	0,152
2015	0,5643	0,3666	0,58350664	0,208182286	0,524901439	0,296559057	0,398373726	0,430994505	0,312404568	0,613082514	0,733	0,1845	0,547144461	0,313117296	0,495707154	0,336284536	0,60130351	0,200047355	0,585	0,211	0,728	0,159
2016	0,5801	0,3742	0,610370527	0,216479697	0,549962003	0,274198964	0,412092067	0,423978465	0,325397301	0,602278861	0,7597	0,1589	0,562117409	0,300958188	0,51826243	0,314282697	0,61542447	0,191480503	0,596	0,192	0,736	0,15
2017	0,5791	0,3762	0,59920837	0,217831163	0,56978963	0,252436257	0,413796909	0,424365342	0,336379178	0,590284737	0,7609	0,1553	0,554817338	0,300579395	0,52532568	0,309388054	0,606462698	0,196411367	0,602	0,197	0,733	0,154
2018	0,5795	0,3761	0,602169779	0,216443235	0,597383127	0,236824172	0,422226889	0,419260854	0,344009695	0,58904663	0,7627	0,151	0,562285548	0,294433448	0,531921062	0,294482729	0,611756251	0,199971435	0,598	0,192	0,727	0,152
2019	0,5819	0,3739	0,60382983	0,215436372	0,614743829	0,224137347	0,435214616	0,40963584	0,350751732	0,583816656	0,7653	0,1435	0,560807712	0,289064256	0,535333659	0,283010597	0,612668997	0,197157182	0,604	0,186	0,726	0,148
2020	0,5867	0,3690	0,624905447	0,207936874	0,634607079	0,214095703	0,501865828	0,354192872	0,356590859	0,57810775	0,7215	0,1831	0,519497968	0,334900646	0,433569716	0,375954068	0,594612757	0,221866872	0,608	0,179	0,739	0,135
2021	0,5869	0,3691	0,61970868	0,21141703	0,63383803	0,214149384	0,49769245	0,3588292	0,367708895	0,568140162	0,736	0,1672	0,518475936	0,3237539	0,465824237	0,334379373	0,586948997	0,220331553	0,584	0,2	0,722	0,147
2022	0,5884	0,3678	0,622476067	0,210034708	0,640959557	0,209129924	0,494642162	0,361526011	0,370623399	0,564421435	0,7572	0,1507	0,539693403	0,306324178	0,519249187	0,294192029	0,601231094	0,206080265	0,601	0,183	0,744	0,131
